# A scoping review of ethical decisions and decision tools for experimental animal protocols

**DOI:** 10.1186/s12910-025-01297-z

**Published:** 2025-11-14

**Authors:** David Mawufemor Azilagbetor, David Shaw, Jens Gaab, Rosa Maria Cajiga Morales, Bernice Simone Elger, Lester Darryl Geneviève

**Affiliations:** 1 Institute for Biomedical Ethics, University of Basel, Bernoullistrasse 28, Basel, 4056 Switzerland; 2 Division of Clinical Psychology and Psychotherapy, Faculty of Psychology, University of Basel, Basel, Switzerland; 3 Care and Public Health Research Institute, Universiteit Maastricht, Maastricht, The Netherlands; 4 Faculty of Social Sciences, Business and Economics, and Law, Åbo Akademi University, Turku, Finland; 5 University Center of Legal Medicine, University of Geneva, Geneva, Switzerland; 6 Faculty of Medicine, Laval University, Quebec, Canada; 7 VITAM – Research Center on Sustainable Health, Integrated University Health and Social Services Center of Capitale-Nationale, Laval University, Quebec, Canada; 8 Quebec Excellence Center on Ageing (CEVQ), Integrated University Health and Social Services Center of Capitale-Nationale, Laval University, Quebec, Canada

**Keywords:** Animal experimentation, Harm-benefit analysis, Weighing of interests, Animal ethics committees, IACUCs, Decision support tools

## Abstract

**Background:**

Scientific research projects involving animals are required to undergo ethical evaluation, generally known as *harm-benefit analysis (HBA)*, to ensure that they address important ethical concerns related to animal welfare and the scientific quality of the research to maximize the likelihood of their potential benefits. Research continuously shows the challenges encountered by decision-makers, prompting researchers to review how HBA is conducted and to propose tools to aid decision-making. However, the extent to which such resources are currently available, their jurisdictions of applicability, and how they guide decision-making are not entirely clear.

**Method:**

Through a Scoping Review methodology, a systematic literature search was conducted in PubMed, Scopus and Web of Science for publications in Europe and North America (USA and Canada) from 1985 to 2023. Title and abstract, full-text, and reference screenings, followed by data charting, respectively, were carried out for retrieved publications using pre-developed and registered review protocol.

**Results:**

17 resources that can guide HBA and decision-making were identified. They discussed what should constitute harm to animals and benefits of research, and how these two interests can be balanced to make a decision. Some adopt mathematical calculations, some propose guidelines for committee discussions, while others propose the combination of different approaches to decision-making.

**Conclusions:**

Decision-making based on deliberation among committee members should be supported over the use of scoring approaches. Additionally, making ethical decisions on a case-by-case basis is preferable to accuracy, which may not be realistically practicable.

**Supplementary Information:**

The online version contains supplementary material available at 10.1186/s12910-025-01297-z.

## Background

Biomedical research involving non-human animals (henceforth animals) has been instrumental in the development of knowledge and its application in biomedicine. It contributes to improving the health and wellbeing of humans, animals, and the environment at large [1, 2]. Nonetheless, it has been understood that the increasing awareness and understanding of animal sentience (and ensuing animal welfare concerns) challenge the need to pursue scientific advancement through animal experiments [3, 4]. Further, the long-standing societal concerns about animals used in research also requires scientists to promote animal welfare in their research endeavors.

In 1959, William Russell and Rex Burch introduced the principles of the three Rs (Replacement, Reduction and Refinement [3Rs]) in their seminal publication, *The Principles of Humane Experimental Technique* [5]. In this work, they proposed the 3Rs to address the ethical and scientific conflicts of animal experimentation, by replacing animals with other methods not involving sentient vertebrates whenever reasonable, possible and appropriate (replacement), reducing the number of animals used whilst maximising data obtained (reduction) and refining experimental procedures to limit harm and suffering caused to animals (refinement) [6]. These principles are to be considered by scientists in designing and performing experiments involving animals. Meanwhile, animal research also raises other challenges in the scientific community. One significant argument is about the reproducibility of the results obtained from animal experiments. Results of animal experiments are often difficult to reproduce, either in the original labs or by other researchers [7, 8]. Some interpret this as a result of poor reporting, poor experimental designs and poor statistical analyses, and over-interpretation of results among other problems [9–13]. Others attribute poor reproducibility to the choice of animal models and environmental conditions [12, 13]. Further, animal research faces other concerns, such as the limited translatability of animal research findings into clinical human health benefits [14–16]. Considering scientific validity in animal research oversight continues to be an important subject [17–20].

These challenges continue to highlight the essential nature of ethical oversight of animal research to ensure rigorous, responsible and ethical conduct of animal experiments. Generally, it is ethically justified to involve animals in research if there are no alternatives, if animal welfare concerns are addressed satisfactorily, and if the experiment will be beneficial to science and society [21] and these benefits outweigh the harm/risks to the animals involved. In this regard, an evaluation of animal experiments known as *harm-benefit analysis (HBA)* is carried out to weigh the harms inflicted to animals against the benefits derived from these experiments. It is generally required that animal research protocols are submitted for this evaluation by oversight bodies [22]. These bodies are mandated to evaluate the scientific relevance of experiments and animal welfare issues, and subsequently decide whether the animal research protocol should be approved [23, 24]. The acceptability of a particular experiment is typically evaluated on a case-by-case basis [25].

The conduct of HBA in practice is not always straightforward, and decision-making through HBA can be challenging, one reason being the fact that the items involved – harm and benefit – are not measurable in the same units [26]. Inconsistent evaluation of research protocols is one of the consequences of this challenge. For example, research reports the occurrence of inconsistencies in the weighing of interests in animal research protocols and in the decision-making of oversight bodies and their individual members [23, 25, 27–31].

To assist in weighing the value of research against animal welfare issues, Patrick Bateson published, in 1986, one of the first aids to be used to determine “when to experiment on animals” [32]. This decision support scheme has been used and reproduced over the years [33]. Meanwhile, how animal experiments should be evaluated for ethical approval is evolving and the continuous reporting of issues regarding the review process and the conduct of HBA [19, 23, 34–36] prompts the need for ways of improving the decision-making process [35, 37]. Authors, working groups, expert panels and workshop programs have, and continue to review how HBA is conducted to propose decision-making aids to foster systematic and consistent conduct of HBA [2, 33, 38–40]. Most authors note some of the existing decision support tools when discussing the decision process or when proposing new aids for decision-making [2, 25, 39, 41, 42]. However, it is not clear to what extent such resources, in the form of guidelines or tools, have been provided or exist in the literature to support decision-making in a more elaborative and systematic manner.

The ethical evaluation of animal experiment protocols involves assessing each protocol against a set of criteria to be considered in decision-making. It would therefore be expected that proposed tools to aid this evaluation will take these criteria into account. However, the exact criteria considered in developing the proposed decision support systems, and their general outlook have not been clearly mapped out. Further, when it comes to the subject of harm, there are various outlooks and perspectives with regards to what should count as harm to animals in experiments [2]. Moreover, the conduct of animal research and the field of laboratory animal welfare science are rapidly evolving and new concepts continue to emerge regarding experimental animal harms and refinement approaches [43–46]. The issue of harm, in addition to what exactly should be considered as harm in animal experiment protocols, is also constantly changing. What is not known is what exactly these decision support tools consider as harm to animals involved in experiments, and how the tools aid their assessment. It is not clear how the outlooks of harm, the assessment of harm, and its consideration in the decision-making process differ from one decision support tool to another. Moving to the category of benefits, as with harm, it is also not clearly discussed what benefits these tools consider a justification for harm, and how these benefits can be assessed in ethics applications. How the criteria of benefit are treated and considered in the overall decision-making process, and how the balancing act differs from one tool to another is also not mapped and discussed in the literature. In essence, how much information provided on the criteria in each of these decision support tools to aid decision-making is not known.

It is therefore necessary to map the contents of these tools to understand the criteria the authors envision in weighing the interests of animal experiments. The resources provided to aid decision-making may be in different forms, go by different names, and/or take different approaches to HBA [2, 41]. Regarding approaches to the HBA process, there are a number of models for conducting HBA. The discourse approach to project reviews and the conduct of HBA involves dynamic and flexible discussion among a group of people with different areas of expertise. This model is therefore centered on ethics committee deliberations on the value of specific research projects while considering harm caused to animals [41]. In addition, there are a number of models that take a more systematic approach to comparing and weighing harms and benefits. Some of these models propose the use of algorithms and mathematical scores to guide protocol assessment and decision-making [2, 47, 48], while others suggest the use of graphic matrices or diagrams to represent and visualize harms and benefits to assist in situating a HBA decision [25, 32, 38]. Still, others propose the use of checklists categorizing key questions that should be answered and key topics that should be explored when making a decision [2, 49]. All these different approaches have their individual strengths and limitations. The discourse approach ensures that ethical judgements are validated through social dialogue; however, decision-making may be non-transparent and inconsistent across projects [41]. On the other hand, metric and structured models provide standardized and harmonized evaluation across projects, but are often criticized for reducing ethical concerns to mathematical calculus and giving a false sense of objectivity in project evaluation [2, 38, 42, 49]. As a result of a need for continuous improvement in consistent decision-making, it is important to map the HBA approaches of the decision support tools available in the literature.

Moreover, these resources originate from different systems [39, 50], and the exact context and jurisdictions in which they can be applied still remain unknown. It is therefore important to map the specific aims and objectives underpinning the proposal of these decision support tools to understand in which contexts they may be applicable. Therefore, via a scoping review, we sought to systematically map the literature on HBA to bring to light the various forms of resources or tools that have been proposed and that could aid HBA and improve the ethical evaluation of animal research project. We started out with the research question: *what strategies exist in the form of decision support systems for the conduct of HBA in animal experiment project evaluations and authorizations*? Sub-objectives of the review regards mapping the literature on the profiles of the proponents of these decision tools, their definitions of harms and benefits and examples provided, the approach proposed to reach decisions, declared jurisdictions of applicability, perceived advantages and disadvantages of the tools, and their contribution to consistent project evaluation.

## Methods

### Scoping review protocol and registration

A scoping review protocol conforming to the *PRISMA Extension for Scoping Reviews (PRISMA-ScR)* [51] was developed and prospectively registered on *Open Science Framework* on April 25, 2023. The full protocol was made publicly available to ensure transparency [52]. The reporting of results of the review in this manuscript adheres to the PRISMA Checklist for reporting Scoping Reviews (see Supplementary File 4).

### Search strategy and information sources

A search strategy was developed for the scoping review in consultation with an information specialist from the University of Basel Library. The information specialist checked the search terms, verified the search string, and helped in finalizing the search strategy to ensure an optimum inclusion of targeted evidence sources. Three electronic literature databases were searched: PubMed, Web of Science (all databases), and Scopus. To achieve our research objective, we considered a broad range of evidence sources, including academic researchers, reports from workshops or published by working groups and institutional committees, since these are the various working approaches through which decision aids for HBA are developed [2, 25, 33, 38, 48, 49, 53]. The final search string used in PubMed is provided in Supplementary File 1 (Search Strategy for PubMed).

#### Eligibility criteria

We included evidence sources addressing the improvement of ethical evaluation of animal research protocols by recommending or proposing decision aids for systematic project evaluations. Comprehensive guidelines that could aid systematic and consistent conduct of HBA were also included from sources found through our search strategy. Regarding the publication languages, only publications in English and French were included, and this was based on the language proficiency of the review team. For the geographical scope, publications from the European Union, the United Kingdom, and North America (USA and Canada) were included, since most publications proposing decision aids come from these jurisdictions, and major working groups for the improvement of HBA have been constituted by members across these contexts [2, 33, 38].

Furthermore, we included publications from January 1985 to October 2023. We included studies as far back as 1985 since one of the first known decision aids proposed for a systematic HBA, *Bateson’s cube*, was published in 1986 [32]. This allowed us to have an extended coverage in our search strategy to include all possible sources published from that time in our scoping review. The initial search was conducted on March 14, 2023 to retrieve publications from January 1985 up to December 2022. The search was repeated on November 16, 2023 to find and include additional works that might have been published from January 2023 to October 2023.

Publications were excluded if they discuss the need to improve the ethical evaluation of animal research protocols without explicitly proposing decision aids, guidelines, or directives for the conduct of HBA. Indeed, the limitations of HBA and the subsequent need for better ethical and consistent decision-making are extensively discussed in the literature [26, 36, 54, 55]. However, most of these discussions were conceptual or theoretical in nature, and most of them do not propose workable tools for the purpose of our review. We therefore focused only on those papers proposing tools designed to achieve ethical and consistent conduct of HBA.

#### Management of search results

After the literature search, results were uploaded to Covidence (covidence.org), a literature manager that aids in coordinating review projects. Covidence helped with the detection and removal of duplicates, the publication screening and selection, design of flow diagram, and the general organization of the review process between review authors.

#### Selection of documents

A publication selection form was developed and calibrated a priori by review authors as part of the development of the review protocol. In the calibration process, three review authors (DMA, RMC, and DS) independently screened the first forty publications of search results. Where necessary, further adjustments were made by the three authors to improve the form. Where there was no consensus between the three review authors, the help of an arbitrator (LDG) was sought. The final version of the publication selection form was approved by all review authors.

When the search results were imported into Covidence, duplicates were automatically detected and removed. The removed duplicates were manually checked against their second copies independently by two review authors (DMA and RMC) to ensure that they were indeed duplicates. During the title-and-abstract screening phase, further duplicates that were not detected by Covidence were detected, marked and removed.

The title-and-abstract screening of publications was performed independently by two review authors (DMA and RMC) based on the inclusion criteria stipulated in the review protocol. The Covidence platform helped review authors to keep track of agreements and disagreements during the selection process. Variations and discrepancies in publication selection between the two review authors were solved through discussions. Where this was not successful, the intervention of one of the two arbitrators (DS and LDG) was sought to reach a consensus.

The subsequent full-text screening of publications was carried out by two review authors (DMA and RMC). Reasons for excluding publications were documented. Discrepancies were resolved between the two review authors through discussions or via the help of an arbitrator (DS/LDG/BSE). Reference screening of included publications was performed and additional publications which met the inclusion criteria were added to the final publications included in the review.

#### The data charting process

To ensure the reliability, consistency, and comprehensiveness of the data charting process, a standard data charting table based on the data items stipulated in the review protocol was created. Independently, two review authors (DMA and RMC) pilot-tested the form by reviewing five evidence sources included from full-text screening. This permitted comprehensive detailing of the variables and data items of interest. Further adjustments, where necessary, were made in an iterative manner by the help of another review author (LDG) to improve the table. The final version of the data charting table was approved by all review authors. During the data charting process, the authors refined the data items, where necessary, to specify the details of each item according to the varying ways by which the authors of each decision aid approached the issue to ensure coherent data charting from the various papers included in the review. The final data charting table is provided in Supplementary File 1 (Data Charting Table). The data charting table allowed review authors (DMA and LDG) to chart data independently.

#### Data items

Data items in the data charting table included publication characteristics: Author(s); Year of publication; Aim or purpose of the publication; Country and region of decision aids; Jurisdictions (national and supra-national) of the applicability of decision aid (UK, EU or North America); Profile of authors of the decision aid (Academia, National legislation etc.); and Approach to HBA process (deliberative or discourse, metric or scoring, graphic representation, etc.). Country and region of the decision aids were determined by the country and regions in which the authors’ institutional affiliations are located. The jurisdictions of the applicability of decision aids were determined by the laws referenced in the publications in addition to the authors mentioning this clearly in the publications.

In addition, data on HBA was also charted, and included:

- Harm: Definitions; Types of harm; What constituents harm according to the authors; Examples provided; Methods and tools for harm assessment; Harm severity categorizations; and Harm severity delineations.
- Benefit: Definitions; What constituents benefit; Areas of interest considered in animal research; Provision of examples; Methods and tools for the assessment of benefits; Classifications of the significance of benefits; and Benefit significance delineations.
- Harm-benefit balancing: How is harm balanced with benefit to reach a final conclusion? What tools are provided for this balancing act? Notes on the applicability of the tools by oversight bodies; and advantages and limitations disclosed by the authors.
- Consistency: How do the decision-aids allow for a consistent evaluation? What are their limitations? Have some of them led to decisional conflict?

Independent outcomes of the data charting process were compared by the two review authors who charted the data independently (DMA and LDG). Discrepancies in the content of the extracted items were discussed and resolved by DMA and LDG with the help of a third review author (DS/BSE).

#### Synthesizing the results

We grouped the results according to the rationale and contexts of the proposition of the decision aids. We summarized the different contexts of application foreseen by the authors of the aids. We also grouped the decision aids according to the approaches they take to reach final HBA decisions. We further mapped each of the documents and grouped the contents, in a tabular form, based on the sub data items of harm, benefits and HBA which are of interest for our review.

## Results

### Selection of documents

**Fig. 1:**
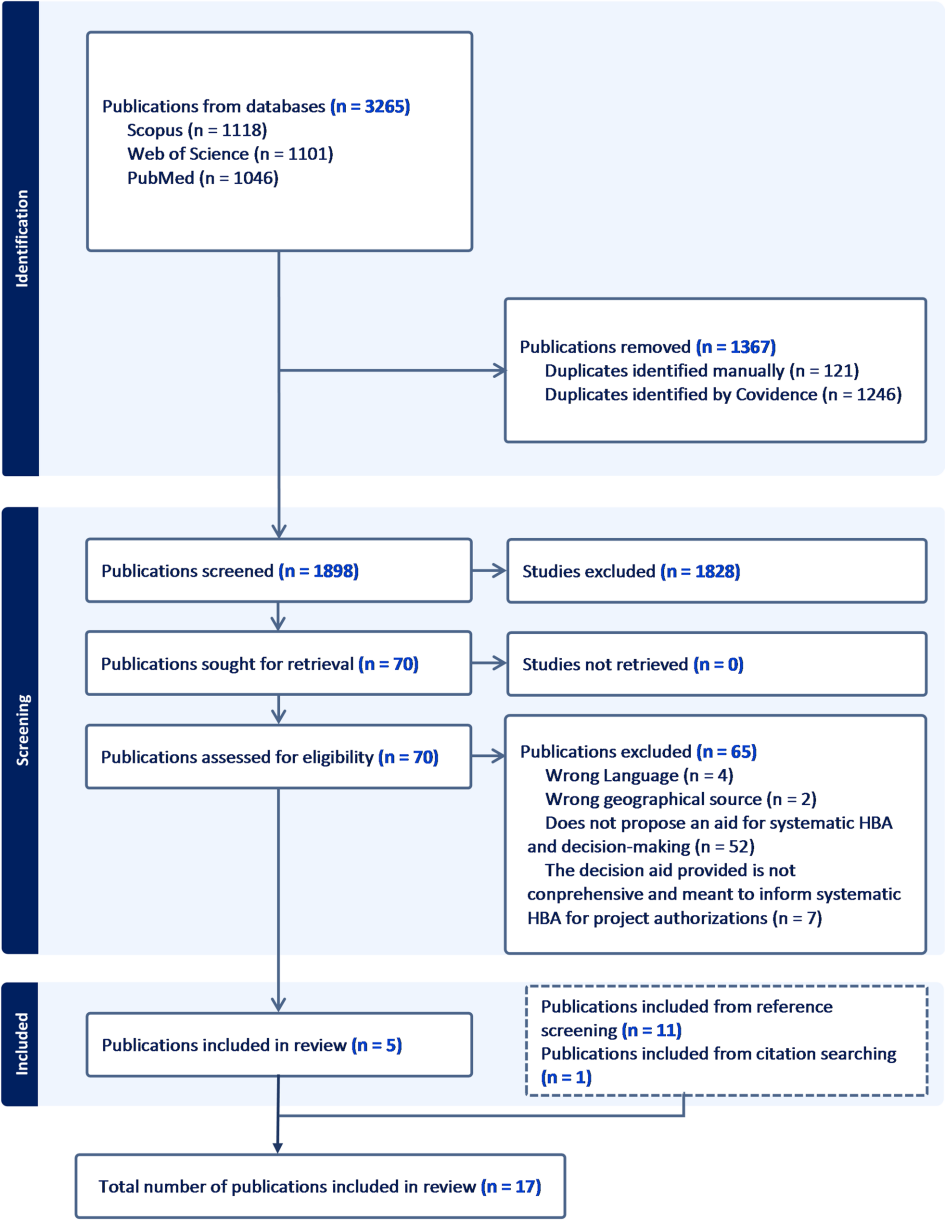
Flow diagram of document selection

Our initial search on March 14, 2023 returned 3,121 search results (PubMed = 1,011; Scopus = 1,053; and Web of Science = 1,057). The updated search on November 16, 2023 returned 144 search results (PubMed = 35; Scopus = 65; and Web of Science = 44). Taken together, search results from the three electronic databases returned a total of 3,265 sources of evidence (See Fig. 1).

A total of 1,367 duplicates were detected (1,246 were detected by Covidence, which were manually verified before removal by review authors DMA and RMC, and 121 additional duplicates were manually detected and removed by review authors DMA and RMC). Consequently, 1,898 sources of evidence were then included in title and abstract screening (Fig. 1). After title and abstract screening, 70 publications were retained and considered for full text screening while 1828 were excluded. At the full text screening stage, 5 publications which met the inclusion criteria were retained and were included in the review while 65 were excluded (Fig. 1).

We conducted a comprehensive screening of the references of relevant publications considered for full-text screening, as well as all the references of the five publications included from the full-text screening stage. This comprehensive assessment led to the identification of 11 documents that met our inclusion criteria, and were included. These 11 documents generally were preceding decision aids that were referenced by authors when they were proposing additional aids or when they were discussing ways of improving decision-making. Furthermore, we also identified one additional decision aid from further citation searching. Thus, a total of 17 publications [25, 32, 33, 38, 39, 42, 47–50, 53, 56–61] were finally included in our scoping review, and went into data charting.

### Results on data items

#### Characteristics of included publications

An overview of the characteristics of each publication is provided in Supplementary File 2. Out of the 17 documents included, 14 were generally aimed at guiding decision-making on any type of animal experiment while two [42, 60] specifically included experiments involving transgenic animals and one [39] included experiments involving tissue engineering. Only one of the publications [53] had a clear aim of proposing an approach to improve the consistency of project evaluations. Three of the publications [33, 38, 56] were products of working groups (or working parties). Of these, one [33] was established by a regional authority (the EU) to facilitate the implementation of project evaluation and authorization through HBA, one [38] was conveyed by professional societies related to animal research, and one [56] was a working party of a charitable organization.

One publication [58] was a report by a statutory independent body with a legal mandate to advise on matters of animal experimentation and study the regulation of animal experiments, while two [49, 61] were reports resulting from research workshops aimed at project evaluations and decision-making. The paper of de Cock Buning and Theune [50] compared three decision aids but only one, the “Dutch model”, which was presented in detail, was charted from this source. The other two decision aids [47, 56] were charted from their full-text documents.

The countries and regions of the authorship of the decision aids as well as the countries and jurisdictions of their applicability are presented in Table 1 (Demographics of decision aids). Eleven of the decision aids [25, 32, 33, 42, 48–50, 56, 58, 60, 61] are uniquely authored from European countries, or referenced European Union or national legislation. Four of them [47, 53, 57, 59] are uniquely authored from North America (USA and Canada), while two of them [38, 39] are collectively authored from Europe and North/South America. Five of the publications specifically discuss the jurisdiction of the possible application of their decision aids in European countries [32, 33, 50, 58, 61] while three of the decision aids [53, 57, 59] are specified for possible applications in the USA and Canada. Two are joint works from Europe and America and may be applied in both regions [38, 39].

**Table 1:** Demographics of decision aids

Decision Aid (Authors/Year)	Country (Author Affiliation/Legislation Referenced)	Region of Authorship	Country of Application	Region of Application
Bateson, 1986 [32]	UK	Europe	UK	Not mentioned
Prentice et al., 1990 [53]	USA	North America	USA	N/A
Smith & Boyd, 1991 [56]	UK	Europe	Not specified	Not specified
Porter, 1992 [47]	Canada	North America	Not stated	Not stated
Boisvert & Porter, 1993 [57]	Canada	North America	Canada	N/A
de Cock Buning & Theune, 1994 [50] (Dutch model)	Netherlands	Europe	Netherlands	N/A
Animal Procedures Committee, 1994 [58]	UK	Europe	UK	N/A
Boisvert & Porter, 1995 [59]	Canada	North America	Canada	N/A
Delpire et al., 1998 [60]	Italy, UK	Europe	Not specified	Not specified
Delpire et al., 1999[42]	Italy, UK	Europe	Not specified	EU
Stafleu et al., 1999 [48]	Netherlands	Europe	Not stated	Not stated
Voipio et al., 2004 [49]	Denmark, Estonia, Finland, Iceland, Latvia, Lithuania, Norway, Sweden and the Netherlands.	Europe	N/A	N/A
Lindl et al., 2012 [61]	Germany	Europe	Applicable in Germany; German laws were cited.	Potentially applicable to other EU member states.
Bout et al., 2014 [25]	Netherlands	Europe	Not specified	Not specified
Laber et al., 2016 [38]	USA, France, Spain and Norway	North America and Europe	Not stated	Not stated: likely North America and Europe
Liguori et al., 2017 [39]	Netherlands and Brazil	Europe and South America	Not stated	Not stated (However, American and EU legislations were referred to)
EU Expert Working Group, 2018 [33]	Europe	Europe	EU Member States where Directive 2010/63/EU is applicable	EU Member States and Others affected by Directive 2010/63/EU

In the following parts of our Results section, we will present our results on the variables of harm and benefit and the applicability of the decision aids in the evaluation of proposed projects.

### Harm

#### Definitions of harm

Only one of the papers [38] specifically considered a definition for harm. In this paper, Laber et al. [38] defined harm within the context of any negative impact on five freedoms as suggested by Mellor [62, 63]. The five freedoms include: (i) freedom from pain/injury, (ii) freedom from fear/distress, (iii) freedom from thirst/hunger, (iv) freedom to express normal behavior, and (v) freedom from discomfort or appropriate husbandry.

#### Constituents of harm

Since almost all the documents included in our review did not specifically define harm, we collected what they viewed or considered as constituents of harm done to animals in experiments. Fifteen of the publications [32, 33, 39, 42, 47–50, 53, 56–61] discussed what they consider as harm. Four of these publications [32, 42, 48, 49] viewed animal suffering as a constituent of harm, eight [39, 47, 49, 53, 56, 57, 59, 61] included pain and distress as harm, and four [39, 47, 50, 53] included discomfort as harm to animals. For publications like [42, 48, 49, 56], suffering, pain, distress and discomfort were not noted in the constituents of harm, but rather, in variables such as the examples of harm, types of harm, the assessment of harm, and harm severity categorizations, which we will present in the next sections of our results. Meanwhile, for Laber et al. [38], the constituents of pain, distress and discomfort appear in the definition of harm they considered from the five freedoms which Mellor had adapted to harm description in animal research.

Five publications [49, 53, 56, 58, 61] regarded harm as (ethical) costs of animal experimentation. The publication of Lindl et al. [61], considered negative results from experiments as harm, the only paper to have done so. Additionally, Prentice et al. [53] also considered morbidity/mortality to be experienced by experimental animals as part of harm. The constituents of harm for all the sources included in our review are given in Table 2.

#### Examples of harm

Four sources of evidence [32, 42, 48, 59] listed specific examples of harm beyond the general constituents of harm. Bateson [32] for example, gave the isolation of an animal from other members of its kind in the course of experiments as an example of harm, while Boisvert and Porter [59] listed captivity as a potential cause of chronic stress as an example. Two of the publications [38, 56] discussed detailed case examples of animal experiments which involve harm. Data charted on the examples of harm from all publications are provided in Table 2.

#### Types of harm

Only two publications [39, 48] classified the types of harm to consider in animal experimentation. The types of harm considered by Stafleu et al. [48] were predicted pain and discomfort, intrinsic value, and psychological complexity, while Liguori and colleagues [39] classified them as: physical and psychosomatic harm resulting from emotional distress.

#### Assessments of harm

All publications but two [32, 39] mentioned the act of assessing harm in animal research protocols in their decision aids. As guidelines to be used in assessing harm, two of the papers [25, 61] that considered harm assessment and were published after 2010 made reference to Directive 2010/63/EU [22] while one publication [33] referred to an EU Expert Working Group Reports on Severity Classification and Retrospective Reporting. Specifically for pain assessment, Porter [47] referred to the report of Laboratory Animal Science Association (LASA) working party [64]. The way in which each publication approached how harm should be assessed in proposed animal research projects is summarized in Table 2.

#### Harm severity levels and delineations

All publications but three [53, 59, 60] discussed various levels of harm severity. For the publications that discussed it, five of them [32, 39, 49, 50, 56] categorized harm severity in three-point levels: Low/Minimal/Minor/Mild; Intermediate/Medium/Moderate; and High/Severe/Substantial. In addition to these three levels, the publications of Animal Procedures Committee [58] and Delpire and colleagues [42] had a fourth severity level named “Unclassified”, while the fourth level for Lindl et al. [61] and Bout et al., [25] is “Non-recovery”, based on Directive 2010/63/EU [22]. For Laber et al. [38] harm severity is rather categorized in five levels; the above three categories in addition to “no impact” and “moderate to severe” levels. For Porter [47], Boisvert and Porter [57], and Stafleu et al. [48], harm items are scored to determine their severity levels.

With regard to how the various harm severity levels should be delineated, seven [25, 38, 39, 42, 47, 48, 61] of the fourteen publications that discussed severity levels clearly provided guidelines that can be followed to aid the categorizations. Discussions in Voipio et al. [49] suggested that harm or cost categorization is subjective. With the recommendations given in the decision aid of de Cock Buning and Theune (Dutch model) [50], delineating harm severity levels can be determined through committee discussions. The discussions of Smith and Boyd [56] suggested that the assessment and weighing of costs can be done on case-by-case basis. Finally, Boisvert and Porter [57] proposed that scoring pain and distress should be done anthropomorphically. How harm severity levels can be delineated was not found for the remaining three [32, 33, 58] decision aids. Data charted on harm severity levels and how they can be delineated for each of the publications included in our review are provided in Table 2.

**Table 2:** Harm: definitions, constituents, examples, types, assessments, and severity levels

Decision Aid (Authors/Year)	Definition of Harm	What Constituents (or is viewed in terms of) Harm?	Examples of Harm	Types of Harm	Assessing Harm	Harm Severity Levels	Delineating Harm Severity Levels
Bateson, 1986 [32]	No specific definition given	Animal suffering	Isolating an animal from other members of its kind.	Not discussed	Not discussed	Low; Intermediate; High	Not discussed
Prentice et al., 1990 [53]	No specific definition given.	Ethical costs like pain, discomfort, distress and/or morbidity and mortality.	Pain, Discomfort, Distress, Morbidity and Mortality	Not discussed	By considering the guideline’s harm-related questions on: Housing, Procedures, Physical restraints, Pain control, Post-procedure care, and Euthanasia.	No reference to a categorization system for harm severity	N/A
Smith & Boyd, 1991 [56]	No specific definition given	Cost, which involves type of animal and effects of experiment, husbandry and housing conditions, scientific procedures, and provisions for managing adverse effects.	Surgery involving pancreatectomy; splenectomy; Surgery to induce acute pancreatitis; etc.	Not discussed	Via the assessment of the: (1) Quality of facilities and project workers and (2) Severity of effects of husbandry and procedures on animals.	Low; Medium; High	No specific or universal rules, but based on a case-by-case evaluation for assessing or weighing the costs.
Porter, 1992 [47]	No specific definition given.	Pain; Duration of discomfort, distress or anxiety; Duration of experiment; Number of animals used; Quality of animal care.	None given	Not discussed	By assessing factors involving distress, discomfort, or anxiety; Pain; Duration of the experiment with regard to animal life span; Number of animals used; and Scoring the standard of care of animals.	By scoring these categories, from 1 to 5: Pain; Duration of discomfort; Duration of experiment; Number of animals; Quality of animal care.	Pain: The use of a report by the LASA working party; Discomfort: It is subjective; Number of animals: Scoring on a scale of 1 to 5; Duration of experiment: Based on life span calculation; Quality of animal care: Subjective evaluation
Boisvert & Porter, 1993 [57]	No specific definition given	Pain; Distress; Level of sentience of the species involved; Number of animals used; and Quality of care offered to the animals.	None given	Not discussed	Scoring the following categories of the guideline on a scale of 1 to 5: Pain; Duration of Pain; Distress; Duration of Distress. Assessing modulating factors: Species of Animal; Number of Animals; Quality of Animal Care.	Scoring of Pain and Distress separately on five severity levels.	Scoring of pain and distressed should be done anthropomorphically.
de Cock Buning & Theune, 1994 [50]	No specific definition given	Discomfort to animals (relating to the experiment and arising from housing conditions)	None given	Not discussed	By the use of Checklist (B) provided	Minor; Moderate; Severe	Likely to be determined through discussions.
Animal Procedures Committee, 1994 [58]	No specific definition given	Cost or severity	None given	Not discussed	Cost to animals in suffering, which involves adverse effects on animals + (coping strategies and strategies like anesthesia adopted to address adverse effects).	Mild; Moderate; Substantial; Unclassified (for procedures with anesthesia without recovery)	Not discussed
Boisvert & Porter, 1995 [59]	No specific definition given	Pain; Distress; Level of sentience of the species; Number of animals; and Quality of care.	Captivity as a potential cause of chronic stress	Not discussed	By the use of comprehensive descriptions for the harm-related categories (D, E, F, G, H, I and J) in the Checklist/Guideline.	Not discussed	N/A
Delpire et al., 1998 [60]	No specific definition given	Respect for Well-being, Autonomy, and Justice) for animals, consumers and patients, based on three ethical principles of Beauchamp & Childress	None given	Not discussed	Each of the three principles for each stakeholder group are assigned on a scale of − 3 to + 3. (See also Dolan K. (1999)^#^)	Not discussed	N/A
Delpire et al., 1999[42]	No specific definition given	Animal suffering	Insertion of hypodermic needle; laparoscopy under anaesthesia; vascular cannulation; etc.	Not discussed	By the use of the checklist and guidance notes, and giving an overall assessment using the decision-making system.	Mild; Moderate; Severe; Unclassified	Guidelines and examples provided for delineation.
Stafleu et al., 1999 [48]	No specific definition given	Animal experience (suffering); Psychological complexity; Intrinsic value	Administration of substances with serious effects; starvation for more than 24 h; continuous immobilizations, etc.	1. Predicted pain and discomfort 2. Intrinsic value 3. Psychological complexity	By the use of scores. Formula for discomfort is based on actual discomfort, number of animals and duration of experiment. Formula for harm based on animal suffering, intrinsic value and psychological complexity.	Discomfort is scored on five levels while duration of suffering and number of animals are scored on three levels each.	Definitions and examples are provided to delineate the severity levels.
Voipio et al., 2004 [49]	No specific definition given	Costs, which include animal harm such pain, illness, suffering, and distress.	Pain, distress, discomfort, suffering; Duration, frequency, severity; Death	Not discussed	Subjective evaluation of decision-makers.	Mild; Medium; Severe	Subjective categorization of harm (costs).
Lindl et al., 2012 [61]	No specific definition given	Costs, which include harm, pain, or distress caused to animals and/or negative results.	None given	Not discussed	Reference to Directive 2010/63/EU, Annex VIII.	According to Directive 2010/63/EU: Non-recovery; Mild; Moderate; Severe	Based on explanations in Directive 2010/63/EU.
Bout et al., 2014 [25]	No specific definition given	Not discussed	None given	Not discussed	Reference to a common format for submitting information^##^, pursuant to Directive 2010/63/EU.	Reference to Directive 2010/63/EU: Non-recovery; Mild; Moderate; Severe	Reference to the delineation in Directive 2010/63/EU.
Laber et al., 2016 [38]	Negative impact on five freedoms (pain/injury; fear/distress; thirst/hunger; express normal behavior; and discomfort or suitable husbandry).	Not discussed	Examples provided as part of case studies: Viral infection in mice ( Pain/Injury: Severe flu infection), etc.	Not discussed	By using a table where a suggested list of modulating factors can be scored and given a summary color.	For aggravating factors: No impact; Minimal; Mild; Moderate to severe; and Severe. Harm summary: Scoring from - to ++++. Color system: from white to Crimson.	Definitions, explanations and depiction of color and grades are provided for each modulating factor. How exactly to arrive at these categories for every experiment is however not clear, and it can be subjective.
Liguori et al., 2017 [39]	No specific definition given	Pain (discomfort resulting from injury or disease); and distress (a product of pain, anxiety, or fear).	None given	Physical and Psychosomatic (e.g., as the result of emotional distress).	Not discussed	In terms of expected animal harm: Mild; Moderate; Severe	Definitions are provided for each severity level to aid delineation.
EU Expert Working Group, 2018 [33]	No specific definition given	Based on procedures, species/strain/age of animals, number of animals, their fate, husbandry practices, transportation, etc.	None given	Not discussed	Reference to EU Expert Working Group Reports on Severity Classification and Retrospective Reporting (on the European Commission website^###^). Constituents of harm are key issues to include.	Reference to EU report, and from revised Bateson’s cube: Low (green); Intermediate (amber); High (red)	Not discussed

#: Dolan K. Cost–benefit – the balancing act. In: Dolan K, editor. Ethics, animals and science, Oxford: Blackwell Science; 1999, p. 211–43.

##: EU. 2012/707/EU: Commission Implementing Decision of 14 November 2012 establishing a common format for the submission of the information pursuant to Directive 2010/63/EU of the European Parliament and of the Council on the protection of animals used for scientific purposes (notified under document C(2012) 8064) Text with EEA relevance. 2012.

###: Website: http://ec.europa.eu/environment/chemicals/lab_animals/pubs_guidance_en.htm.

### Benefit

#### Definitions of benefit

Only three publications [38, 49, 61] contained specific definitions of benefit. Voipio et al. [49] and Lindl and colleagues [61] considered benefits relating to health care and increased knowledge in their definition, while Laber and colleagues [38] defined benefits in terms of social benefits, socio-economic benefits, scientific benefits, educational benefits, and safety and efficacy. The EU Expert Working Group [33] did not define benefits, but mentioned that benefits should be linked to the purposes of project in Article 5 of Directive 2010/63/EU [22], which are listed under the examples of benefits (see Table 3).

#### Constituents of benefit

In Bateson [32], benefits are viewed mainly in terms of medical benefits, which may be substituted for “good science” for a clear public communication on animal research. For Porter [47], the aims of an experiment are rather considered as constituents. Boisvert and Porter [57, 59] viewed benefit in terms of the overall aims of an experiment in addition to their relationship or relevance to the overall objectives. Four publications [42, 50, 53, 56] regarded benefits in terms of the value or significance of research. The full results of what were viewed as benefits are presented in Table 3.

#### Examples of benefits

Nine of the sources of evidence [33, 38, 39, 42, 48–50, 56, 58] provided specific examples on benefits. Two publications [38, 56] provided comprehensive case examples within which concrete benefits can be discussed. Examples of benefits provided by each publication are included listed and discussed in Table 3.

#### Areas of interest in animal research

Nine publications [25, 33, 38, 39, 42, 49, 50, 60, 61] discussed areas of interest in animal research. They all considered basic research or research for the advancement of scientific knowledge as an area of interest in animal research, while five of them [25, 38, 42, 50, 61] added experiments for the purpose of education and training. Delpire and colleagues [42] added the development of alternative methods and the breeding of animals for experimental purposes as areas of interest. Bout et al. [25] considered forensic research in addition, while Voipio et al. [49] and Laber at al. [38] considered economic benefit as an area of interest of animal research. Comprehensive data charted on the areas of interests considered by each decision aids are provided in Table 3.

#### Assessments of benefit

All publications but two [32, 39] clearly discussed benefit assessment. Smith and Boyd [56] proposed the use of a grading scale to assess benefits while Boisvert and Porter [57] and Stafleu et al. [48] suggested scoring categorical items in assessing benefit. Stafleu et al. [48] noted that health interests weigh more than economic or knowledge interests, and recommends assigning them the maximum score of 10, whereas economic or knowledge interests were assigned a maximum of 5 each. In Delpire et al. [60], benefits are assessed by determining whether the research proposal addresses a significant issue for which no practical method exists and whether the quality of the research is capable of attaining the objective. In the decision aids of Animal Procedures Committee [58] and Bout et al. [25], the assessment of benefits is subjective. Bateson [32] and Liguori et al. [39] did not discuss how benefits should be assessed in their decision aids, with Bateson [32] mentioning that benefits are uncertain. Full data charted on how each of the decision aids approached the assessment of benefits are provided in Table 3.

#### Significance levels of benefits and delineation

Significance levels of benefits were found in only ten decision aids [25, 33, 38, 39, 47–50, 56, 57]. In six of those decision aids [25, 33, 39, 49, 50, 56], the significance of benefit is considerable in any of established three levels: Low/Minor/Small; Medium/Moderate/Intermediate; or High/Great/Large. Meanwhile, Laber and colleagues [38] added two additional significance levels; “neutral” and “no positive impact”. In the decision aid of Stafleu et al. [48], the interest of an experiment for human beings is scored on a scale from 0 to 10 to determine its significance level. For the decision aid of Porter [47, 48, 57], the scoring systems are provided for scoring significant levels of benefit.

When it comes to how the various significant levels of benefits should be delineated, only two of the ten publications [38, 39] that discussed significance levels clearly provided guidelines that can be followed to aid the categorizations. Data charted on the classification of the significance levels of benefits and how these levels can be delineated are provided in Table 3.

**Table 3:** Benefits: definitions, constituents, examples, areas of interest, assessment and significance levels

Decision Aid (Authors/Year)	Definition of Benefit	What Constituents (or is viewed in terms of) Benefit?	Examples of Benefit	Areas of Interest	Assessing Benefit	Levels of Benefit Significance	Delineating Levels of Benefit Significance
Bateson, 1986 [32]	No specific definition given	Medical benefits (quality of research and certainty of benefits); Good science	None given	Not discussed	Not discussed	Not discussed	N/A
Prentice et al., 1990 [53]	No specific definition given	Value of research for: human or animal health; advancement of knowledge; and good of society.	None given	Not discussed	Assessing benefits in terms of their value to the three constituents of benefits.	Not discussed	N/A
Smith & Boyd, 1991 [56]	No specific definition given	Social, scientific, economic, educational and other values.	Potential treatment of human diabetes; Developing effective treatment for human pancreatitis, etc.	Not discussed	Use of a grading scale to assess project’s benefits, quality, validity and necessity of methods, and an overall assessment.	Low; Medium; High	Not discussed
Porter, 1992 [47]	No specific definition given	Alleviating human or non-human pain and suffering; Benefit to health or welfare; Fundamental research to advance knowledge.	None given	Not discussed	Scoring, from 1 to 5, the aim of the experiment and its realistic potential to achieve the objective	By scoring, from 1 to 5, aim of experiment and its realistic potential to achieve objective.	Not discussed; (depends on subjective evaluation)
Boisvert & Porter, 1993 [57]	No specific definition given	Overall objective of an experiment.	None given	Not discussed	Scoring, from 1 to 5, overall objective of experiment, its relation to overall objective, and its realistic potential to achieve its immediate objective.	Ranking experiments between 1 to 5.	Not discussed
de Cock Buning & Theune, 1994 [50]	No specific definition	Significance of the research in areas of human/animal interests: routine research, diagnostics, education, problem oriented research and basic fundamental research.	Production, control or biological standardization of sera, vaccines, etc.; Identifying or detecting diseases or other physical symptoms.; Transfer of knowledge; etc.	Routine research: Diagnostics; Education; Problem-oriented research; Basic scientific research	By the use of the check list (C) provided in the guideline.	Minor; Moderate; Great.	Not discussed (Likely to be determined through discussions)
Animal Procedures Committee, 1994 [58]	No specific definition given	Human, animal and ecological; scientific; educational; economic; and other benefits.	Improved health or welfare, safeguarding of the environment; Increasing scientific knowledge; Forensic enquiries; etc.	Human, animal and ecological; scientific; educational; economic; and other benefits.	By assessing importance of research objectives and probability of achieving them.	Not discussed	N/A
Boisvert & Porter, 1995 [59]	No specific definition given	Overall objective of the experiment	None given	Not discussed	Discussion of overall objective of the experiment, its relation to the overall objective, and the scientific merit.	Not discussed	N/A
Delpire et al., 1998 [60]	No specific definition given	The research must address a significant concern for which no viable alternatives exist	None given	Basic research; Applied research	Scoring, from 0–3, the questions relating to the availability of alternatives and the quality of research.	Not discussed	N/A
Delpire et al., 1999[42]	No specific definition given	Significance/value of research for science, society, economy, and educational.	Increasing scientific knowledge; preventing, diagnosing or treatment disease; regulatory testing; interests of human/animal health and environmental protection; etc.	Basic research; applied research; product development and testing; environmental protection; education; developing alternative methods; breeding animals for research.	By the use of the check list and guidance notes, and giving an overall assessment using the decision-making system.	No significance levels discussed for benefits	N/A
Stafleu et al., 1999 [48]	No specific definition given	Human interests for health, knowledge and economics.	Developing a drug against serious disease; economic interests (for industry, nation, or human welfare); etc.	Health interests; knowledge interests, economic interests	Through the scoring of health interests, knowledge interests and economic interests.	Scoring the interest of the experiment for humans, from 0 to 10.	N/A
Voipio et al., 2004 [49]	Benefits, like therapies and increased knowledge, gained by humans or other larger groups from animal research.	Not discussed	New drugs/therapies; increased quality of life; Safety/toxicology; learning; preservation of wildlife; improved production; increased knowledge; etc.	Human health; animal health; safety (toxicity); increasing knowledge; ecology; economy (macro)	By evaluating the aim of the benefit of the single experiment and benefit of the total process.	High; Moderate; Low	Subjective evaluation based on the aim, probability of reaching aim, and quality of research and research group.
Lindl et al., 2012 [61]	Benefits involve possible knowledge gain of and its potential in treating diseases.	Human, animal, or environment needs, and the pain, suffering, and distress of animals in meeting these needs.	None given	Purposes for translational or applied research; protection of the environment or of species diversity; education or training; and basic research.	Assessment of purpose of the research (translational or basic), likelihood of transferring benefits; and using the decision aid (checklist).	Not discussed	N/A
Bout et al., 2014 [25]	No specific definition given	Benefits for science, society (medical advances, environmental protection, etc.) and training.	None given	Directive 2010/63/EU: Basic and applied research; product testing; environmental protection; preservation of species; forensic research; and training of professional skills	Discussing whether the study addresses a health problem, concerns a local or global issue, is innovative to publish in a prestigious journal, or has immediate or future applications.	Low; Moderate; High.	Subjective categorization by members of the decision-making body.
Laber et al., 2016 [38]	Benefits for societal health; socio-economics; science; education; and safety and efficacy.	Not discussed	Health and economic benefits to gain in researching a viral infection, etc.	Social; socio-economic; scientific; educational; and safety and efficacy benefits.	Assessment via a table listing modulating factors for benefits using color codes or “plus” scores.	High impact; Moderate; Neutral; Minimal; No positive impact	By the use of color and “plus”
Liguori et al., 2017 [39]	No specific definition given	Only human benefits are considered.	Increase in basic biological knowledge, new or better treatments for life- and nonlife-threatening diseases, etc.	Tissue engineering in: Basic and applied research	Not discussed	Small; Medium; Large	Definitions are provided to aid categorization.
EU Expert Working Group, 2018 [33]	Benefits not defined, but they Should be linked to the purposes of project in Article 5 (See examples of benefits)	Not discussed	Increasing knowledge, product safety, a new vaccine to improve health for humans, advancing knowledge in a scientific discipline, etc.	Basic research, safety assessment, and improved human/animal health	By discussing what the benefits will be, who will benefit, how they will benefit (impact) and when (and where possible) benefits be achieved.	From revised Bateson cube: Low (red); Intermediate (amber); High (green).	Not discussed

### Harm-Benefit balancing

#### Approach to HBA

Based on the evaluation of the harm and benefit aspects of proposed animal experiments, a balancing act is carried out to determine which part outweighs the other. Data charted on this balancing act and how decision-makers can arrive at a final decision on each animal experiment are provided in Table 4. This also includes the tools provided for the exercise and authors’ discussion of the applicability of the decision aids.

### Advantages and limitations of decision aids

Data on the advantages and limitations, including the strengthens and weaknesses of the decision aids are provided in Table 4.

### Consistency in Decision-making

Regarding the consistency of decisions made using identified decision aids, only the publication of Prentice and colleagues [53] considered this as an attribute of their decision aid, and reported that the decision aid had led to consistent decision-making. Meanwhile, 5 decision aids [39, 47, 48, 57, 60] that incorporate exact methods such as arithmetic scoring of the aspects of harm and benefit, have the potential to aid consistent decision-making. With regard to whether their decision aids have led to decisional conflict, only the decision aid of Voipio and colleagues [49] provided information. Voipio and colleagues discussed that when participants were asked to review cases at the workshop from which this decision aid emerged, variabilities were observed regarding the scoring of harm, benefits and means. Data on how each decision aid may allow for consistent evaluation and their limitations are provided in Supplementary File 3.

**Table 4:** Harm-benefit balancing: approaches taken, the act of balancing, tools provided for this act, and the applicability of decision aids

Decision Aid (Authors/Year)	Approach to HBA	The Act of Harm-Benefit Balancing	Tools Provided	Application by Decision-makers	Advantages	Limitations
Bateson, 1986 [32]	Graphic representation	Use of a decision cube to assess quality of research, certainty of medical benefit and animal suffering. Projects falling within the opaque section are rejected.	A cube: (Bateson’s cube)	Applicable to UK decision-making bodies.	Acknowledges that multiple factors matter in decision-making	Not discussed
Prentice et al., 1990 [53]	Combined: Categories (protocol review guide) and Discourse (discussion)	Protocol review instrument enables assessment of harm, benefit and the other. Final decision via committee discussion.	A protocol review guide.	Each committee evaluates the investigator’s response to all relevant items.	Not discussed	Ethical rationale for decisions still vary
Smith & Boyd, 1991 [56]	Categories	The decision aid provides items to evaluate. Final decision seems to be based on value judgements by decision-makers.	Two assessment schemes (for benefits and costs)	Not discussed	Not discussed	A difficulty of how judgements about each separate dimension can be combined to reach an overall assessments of costs and benefits.
Porter, 1992 [47]	Scoring/Metric	By scoring the various (integrated) aspects (categories) of harm and benefits using the decision toolkit to arrive at a final score to make a decision.	Scoring system, from 8 to 40.	(1) The tool adopts an animal rights approach to guide scientists to ethically assess their experiments; (2) Usable by committees to evaluate proposed experiments.	(1) Draws the attention of researchers to be objective with factors that affect animals; (2) Advocates for a change in philosophy and challenges researchers to design quality research.	Implies accurate assessment where there can be none.
Boisvert & Porter, 1993 [57]	Scoring/Metric	By adding up the scores from the 10 categories.	A score sheet (via mathematical arithmetic)	To be applied by the Canadian Animal Care Committee (ACC).	Not discussed	(1) Too subjective; (2) Only suitable for research on captive animals; (3) Bias against basic research.
de Cock Buning & Theune, 1994 [50]	Combined (Categories and Algorithm)	Detailed questions on quality of the experiment, discomfort, significance and credentials of the research group, help trace morally significant aspects of a proposal. The checklist offers a visual impression of the overall score. Harm and benefit are balanced using the matrix.	A checklist, decision-tree and balancing matrix (Table)	Committee members need to go through the form to indicate the sections and aspects that would possibly be problematic for them. In a plenary session, members can then focus their discussion on those sections.	(1) Comprehensive checklist and decision tree give general visual impression of the overall score and allow clear evaluation; (2) Its robustness helps to control its internal weaknesses; (3) A properly completed checklist-form might serve in justifying decisions; (4) The checklist might serve as a discussion paper in public debate.	(1) Developed according to particularities of its country of origin; (2) Discrimination between research projects with health or nutrition as a primary interest and projects with these as a secondary interest; (3) Unclear and unjustified decisions for basic research.
Animal Procedures Committee, 1994 [58]	Algorithm/metric (Benefit divided by Cost)	Justification is determined by dividing the benefits (importance and probability of achieving objectives) by the costs (adverse effects and coping strategies).	Mathematical formula for benefit-cost analysis	At the time, it was being used by decision-making bodies in the UK	Not discussed	Not discussed
Boisvert & Porter, 1995 [59]	Discourse model (which can potentially lead to a score or value to benefit/cost)	Checklist with 10 categories, with 3 categories (A to C) attributed to the assessment of benefits and 7 (D to J) to the assessment of costs.	A checklist to guide cost-benefit balancing	Applicable to Canadian ACC: The need for animals must be convincing, and benefit must outweigh cost.	(1) Ethical cost checklist can help committees to focus on protocol deficiencies; (2) It can assist in ensuring equal, equitable and ethical protocol reviews.	Not discussed
Delpire et al., 1998 [60]	Scoring/Metric	Answering two entry-level questions followed by an ethical assessment of impacts on interest groups. The sink ensures that a single significant cost can lead to rejection, regardless of benefits.	A scheme	Potentially applicable in the EU	Gives detailed attention to research projects involving transgenic animals.	Not discussed
Delpire et al., 1999[42]	Combined: Categories (Checklist) and decision-making matrix (Table)	The table (matrix) is completed for the four criteria included in the application form by assigning a decision to each of the criterion.	(1) Matrix; (2) Checklist; (3) Guidance notes; 4)Transgenic data sheet	Tool should be usable in the EU.	(1) Considers and includes a separate information sheet for transgenic animals; (2) Allows a standardized evaluation process throughout the EU; (3) Flexible, and welcomes different attitudes.	Not discussed
Stafleu et al., 1999 [48]	Scoring system	By computing the final scores for human interests (health, knowledge and economic), ultimate aim; relevance experiment; and harm to the interests of the animal.	(1) Scoring system with formulas; (2) Eight-step decision-making algorithm	To be used by decision-making committees.	(1) Checklist covers all morally relevant factors; (2) Assists in finding essential factors underlying a decision and illustrates the consequences of alternative decisions; (3) Presents specific ethical decisions and assumptions.	(1) Developed mainly for medical research; experiments education purposes, veterinary science not considered. (2) Criticized for putting metrics in ethics.
Voipio et al., 2004 [49]	Combined: Graphic representation (Modified Bateson’s cube); Categories (Harm and Benefits); and Discourse	Conducting a *cost-benefit-means* analysis. Evaluating means when costs are medium-severe, benefits are moderate-high, or when costs are higher than benefits. Severity degrees preferred over mathematical calculations.	Modified two-dimension Bateson cube.	Potentially applicable by decision-makers, but further debate needed to decide on the level of detail in the application forms, which may depend on the situation.	(1) Long checklists for benefits and costs helps in seeing the overall picture and provides awareness of all possible costs and benefits (2) Short checklists make evaluation easier and helps to focus on the main points.	(1) Long checklists create large inter-individual variation; (2) Short checklists risk comprehensive overview and increases chances for incorrect judgement.
Lindl et al., 2012 [61]	Discourse with Categories (Checklist)	Answering questions (in categories) stipulated in the application form.	An application form (checklist) with categories	Decision-making bodies can apply the form as criteria for evaluation.	(1) A practical guide for assessing and structuring various aspects of animal research applications; (2) Checklist represents a step in recognizing unjustifiable proposals, relevant severity level, and scientific value.	Not discussed
Bout et al., 2014 [25]	Graphical representation with a discourse approach	The use of a graphical matrix showing the balance between different levels of harm and benefit.	A matrix	Usable by committees as descriptive illustration (not a statutory framework). To enhance debate of the justification of animal research and facilitate dialogue between committees and researchers.	(1) Combines normative and procedural models, making ethical reasoning explicit and resulting judgments consistent, realistic and explainable; (2) Enhances communication between committees and the researchers; (3) Helps researchers and committees to describe benefits more explicitly.	(1) Limited in the assessing scientific and societal benefits; (2) Does not address the ethics of animal research or research in general.
Laber et al., 2016 [38]	Combined: Scoring system (numeric) and Color scores (graphic representation)	The use of a template to assess benefits and harms, modulating factors (MF); and their mitigating and aggravating effects using a summary color and “plus” scores.	Template (table) used with “plus” and color codes.	To be used by decision-making committees.	(1) Template helps to standardize the assessment approach; (2) Provides a list of definitions for each modulating factor; (3) Helps to conduct a broad, inclusive and transparent HBA.	Not discussed
Liguori et al., 2017 [39]	Combined: Algorithm (metric) and graphical representation	By grading human benefits versus animal harm while visualizing harm, benefits, and translational gap to reach a final score for a decision.	Algorithmic flowchart; Graphical tool; Scoring table.	Usable by committees and investigators as it considers tissue engineering (TE).	(1) Has a scientifically rigorous and comprehensive background; (2) Incorporates a TE component often missing in other tools.	The selection animal model plays a role and may render the application of the tool difficult.
EU Expert Working Group, 2018 [33]	Graphic representation (revised Bateson’s cube)	Colored cubes depict links between the harms, benefits and likelihood of success: Green cubes depict a favorable HBA while detailed analyses are needed for amber and red cubes.	Revised Bateson’s cube.	Applicable by EU Member States.	The revised cube is applicable in already existing frameworks for project evaluations.	A checklist is still needed to cover all issues in the evaluation.

## Discussion

Our scoping review mapped existing literature on available tools and decision aids for the review of scientific projects involving animals. Publications generally regarded harm as pain, discomfort and distress to animals, although most (all except one) did not give a definition of harm. Benefits of animal research are generally related to the alleviation of human or non-human pain and suffering, protection of the environment, the advancement of knowledge, and education and training. Some of the tools took a mathematical approach to decision-making while others proposed the use of elaborate checklists to guide discussion among oversight committee members. The decision aids were generally intended to guide decision-making on any type of animal experiment, but some also aimed at research involving transgenic animals, tissue engineering research, or improving the consistency of evaluations.

Our scoping review includes publications from Europe (the EU and the UK) and North America (USA and Canada). Based on the number of publications identified, the majority of the decision aids identified in this review were European. This can be explained by the fact that there are more countries or jurisdictions in Europe, with more decision aids adhering to national policies and regulations, as compared to North America, with only two countries (USA and Canada) considered in our review. Additionally, in reaction to the stricter EU Directive of 2010 [22], more decision aids were published to enable its interpretation or implementation, increasing the number of decision aids proposed in Europe. We also observed international collaborations regarding the proposal of the decision aids, which demonstrate collective efforts from different countries to assist and improve the ethical oversight of animal research. The decision aid of Laber et al. [38] for example, is a product of a collaborative working group comprising of the American Association for Laboratory Animal Science (AALAS) and the Federation of European Laboratory Animal Science Associations (FELASA), which aimed at promoting the “common understanding of the principles and approaches to HBA as an important element in the ethical evaluation of the use of animals in the USA and Europe” [2].

The translational relevance of animal models continues to be a major concern in the research community. Many animal studies still lack transparent explanation of their likely benefit to humans. For example, a recent analysis of animal study applications in the Netherlands found that researchers often chose animal models based on the availability of the model and required expertise, rather than on scientific evidence of predictive value for humans [65] (Veening-Griffioen et al. 2021). The translational potential of an animal experiment is strongly related to how beneficial the experiment will be, specifically, how likely the findings will be translatable for clinical applications. There are some tools and frameworks to assist in the selection of the most relevant animal models to increase the translatability of animal research [66, 67]. Translational potential demonstrates an animal experiment’s scientific quality. Scientific quality and the likelihood of results are considered an essential part of the decision process where significant weight can be given to the aim of an experiment. As a result, the translational relevance and potential of animal experiments should be a major component of the HBA [39]. Researchers need to provide clear justifications about how the selected animal model fits the disease and why the model is the most relevant and translatable so evaluators can assess this information [33]. How soon the expected results will be applicable to the benefit domain also needs to be mentioned and evaluated [38]. As discussed by Lindl and colleagues [61], claims about the translational value of an animal model should not be based on speculation, especially for animal research projects classified as translational or applied. Existing scientific literature must support any claim that an animal model qualifies as translational. In our review, only the decision aid proposed by Liguori and colleagues [39] elaborated guidance on how researchers should provide justification of translational potential based on scientific literature to address the translational gap between animal models and humans. The omission of consideration of the translational value of experiments in the other decision aids poses a challenge to an effective conduct of HBA. Future decision aids need to explicitly include translational relevance as a criterion of HBA. Clear guidelines and checklists need to be provided to guide evaluation. This will also drive researchers to select appropriate models, and evaluators can flag proposed animal models with questionable relevance to human biology. Meanwhile, beyond the justification and evaluation of translation relevance, it is also understood that there is an interrelation between the refinement of experiments and bridging the translation gap between animal models and human application, addressing differences between what is observed in animal models and in human patients [39, 68]. Indeed, it is evident that the use of appropriate animal models to ensure effective translation of findings is influenced by species-specific characteristics, environmental conditions, and experimental design. The alignment of these factors in a research setup reinforces refinement, minimising the potential harm to the animals involved, and enhances the scientific validity of the study [39].

Additionally, evaluating whether researchers have considered alternative approaches is an integral part of taking a global approach to assessing benefits [49]. The core principle behind HBA concerns proportionality of harm and benefits. If a non-animal alternative exists to achieve the same benefits, then subjecting animals to harm is unnecessary. As with the case of translational relevance, researchers need to demonstrate how they sought alternative methods for answering their research questions. This necessitates the presentation of evidence indicating thorough consultation with appropriate sources or regulatory authorities, as well as a critical evaluation of relevant information supporting the necessity of animal use [42]. Literature searches and consultations of journals and databases for alternatives should also be documented [38, 50]. While the decision aids generally mention prior consideration and review of the availability of replacement methods as part of the decision-making process, they do not elaborate how this review should be conducted and how decision-makers may ascertain and verify claims about prior search for replacement methods. Future decision aids and guidelines can incorporate prompts like the following for researchers: *What alternatives did you search for? In which databases? What were the results? Is there a validated non-animal test available for this procedure? If no alternatives exist*,* explain why the animal model is necessary and what unique information it provides.* These prompts can assist researchers to effectively seek alternative methods and help evaluators assess researchers’ claim of prior search for these methods.

It was observed that some decision aids were designed to focus on addressing animal interests and harm. Even though the decision aid of Porter [47] can assist decision-making, its focus was to guide scientists in designing ethically acceptable involvement of animals in their research. Generally, the same applies to almost all the decision aids, since they outline what oversight authorities should assess to decide on whether proposals should be accepted, modified or rejected. The availability of these published tools thus creates awareness in the scientific community regarding project evaluations, as scientists can design their experiments ethically, and write their ethics applications accordingly, in a manner that is accessible for decision-makers. This, of course, can have a positive effect on the outcome of their ethics applications. In fact, how scientists should write their animal research ethics applications in order to increase their chances of approval has been the subject of other research [69, 70]. That being said, it can also be observed that some decision aids have the tendency to focus on only one specific side of the harm-benefit analysis, for instance only on animal interests. The objective of Porter’s decision aid [47] for example, was to propose “a simple scoring system to explore experiment from the viewpoint of the research animal”, mentioning that by “adopting the compassionate ‘animal rights’ ideal to guide their experimental studies, scientists would strengthen their ethical position” [47]. For the sake of animal interests, it may be said that research may be designed with animal interests in mind, and focusing initially on animal welfare, followed by the HBA [55]. This is especially the case when it comes to the ideals of animal welfare and the need for humane experimental designs and the application of the 3Rs [20]. But in performing an HBA, it is understood that the interests of both humans and animals need to be weighed to make a decision. As a result, an approach such as Porter’s may be described as tilting the balance towards one side even before the balancing act takes place. Furthermore, animal rights arguments generally do not support the practice of animal research since the interests of animals should not be sacrificed for humans [20]. So, even though it is expected that focusing on the designing and evaluating animal experiments based on animal rights ideals can help to address the polarity of animal research debates and make animal research more acceptable to critiques, this may not actually be the outcome.

These findings indicate differences in the philosophies of the authors proposing decision aids. This observation is not surprising, given that animal research is a subject on which people have different views, even among experts in the scientific and philosophical communities [15, 71–75]. It can therefore be expected that researchers proposing decision aids take different approaches based on different views regarding what should count as harm to animals, inasmuch as the ethical justification of animal experiments is grounded in the requirement that potential benefits must outweigh the harm done to animals. Meanwhile, a decision aid having a specific implicit or explicit ethical outlook (promoting either animal interests or human interests) may have the tendency to influence the evaluation and affect the direction of which experiments will be accepted and which ones will be rejected.

Furthermore, there appears to be a general agreement regarding the main constituents of animal harm, such as pain or suffering, across all decision aids. However, our findings also indicate that some authors consider other factors as harm to animals. For example, Lindl and colleagues [61] consider negative results as harm, Delpire et al. [60] add the biomedical ethics principles of respect for autonomy and justice, and Laber et al. [38] consider actions that impact the five freedoms animals should have. This observation again appears to show differences in views on animal research and its oversight. This is also seen in how the benefit aspects of decision aids are approached. As with harm, proposers of the aids also allotted different levels of importance to the constituents of benefit and the areas of interest. For example, the decision aid of Stafleu and colleagues [48] gives the most significant score of 10 to research of health interest while giving a maximum of 5 points to research projects that advance knowledge and economic interests. This decision aid therefore appears to prioritize medical research. Meanwhile, the tendency of a decision aid to favor applied research in the evaluation process while being biased against basic research is considered a limitation of other decision aids [50, 57]. Approaching animal research in a way that emphasises practical benefits (favoring translational or applied research) over basic research that advance scientific knowledge, has been a major concern of many authors [36, 54, 55, 76–78]. In essence, the view that applied research is more important than basic research appears to be problematic since applied research follows from the basic research that generated the knowledge. Further, Grimm and colleagues [36] were also concerned that implementing the HBA process to focus on practical benefits may erode the credibility of research, which is an investigative endeavor. It would therefore be necessary to appreciate the value of potential knowledge as a primary benefit [2, 38], and prioritize evaluation of this aspect in project reviews rather than focusing on any immediate practical benefits of a particular proposal [36].

Further, an evolution in the concept of weighing of interests in animal research evaluations was observed across time. Earlier decision aids [49, 53, 56, 58, 61] used “cost” to animals but in the later decision aids, “harm” was predominantly used [25, 33, 38, 39]. “Cost” came to be rejected because of its association with economics. It is clear that the use of “cost” here does not refer to economic costs but rather, costs to animals’ welfare or wellbeing. This is perhaps similar to the use of “interests” in the concept of “weighing of interests” where animal interests concern their welfare or wellbeing, whereas human interests concern potential benefits or the general areas of interest in animal research. But it is understandable that there is a wish to avoid the monetary notion that might come to mind when “cost-benefit” is mentioned since cost-benefit analysis is normally conducted for economic purposes. “Harm” makes it clear that it is the negative impact on animals that is being referred to [2]. The HBA terminology is now increasingly used globally for animal research project evaluations. Meanwhile, there are also arguments for the use of “risk” in place of “harm”. Kinter and Johnson [79] for example, argue for the use of “risk-benefit analysis” as opposed to the “harm-benefit analysis” terminology. They argue that the use of “harm” should be avoided since it is misleading and may damage the scientific community as it refers to pain, distress and suffering, even though most animals used in research are not damaged this way due to humane treatments and the application of the 3Rs. They suggest the use of the term “risk” since “risk-benefit analysis” assesses the risk of pain, distress or injury that the animals may experience in research [79]. That being said, should a switch to “risk” be considered, the full terminology should be “risk-potential benefit analysis,” to avoid giving the impression that benefits are certain,

Additionally, insights emerged about striving for the ethical acceptability of decisions rather than accuracy and consistency. We observed that the general aim of these tools was to assist in the evaluation and decision-making on proposed research projects. But the decision aid of Prentice et al. [53] aimed specifically at improving the consistency of decision-making. This finding is important for our review since mapping data on the consistency of decision-making is one of its objectives. The decision aids that adopt metric models to reach final decisions also aim at consistency [47, 48, 57]. Prentice and colleagues [53] argued that it is desirable that decision-making is maximally consistent, but consistency might not prove or guarantee that decision-making bodies are making ethically correct decisions; they could make consistently bad decisions.

As well as consistency, an issue emerged regarding the projection of a sense of objective and accurate evaluation, which is again true of the decision aids using exactness approaches like metrics in decision-making. Arithmetical approaches [47, 48, 57] involve the allocation of scores to the various items considered in the HBA mathematical formula to arrive at a final score that will determine whether the project should be rejected or accepted, in contrast with discourse models which are centered on committees’ discussions and more subjective decision-making. But based on our review, it appeared that the most desirable goal of animal research oversight is not to achieve a sense of objectivity. Participants attending the Nordic-European workshop for the decision aid of Voipio et al. [49] also did not support scoring systems since they can give a false impression of objectivity. The decision aid of Laber and colleagues [38] avoided the use of numbers in scoring benefits, and used “plus” scores and color codes instead. Additionally, in the decision aid of Porter [47] which adopted an exactness method, the authors admitted this limitation of scoring systems. Even though they project a sense of accurate measurement, any such impression is misleading as it is not possible to achieve true accuracy in this context.

These findings highlight the ethical limitations of striving for objective and consistent decision-making rather than ethically acceptable decision-making. Instead of adopting methods that might seem objective, the most desirable goal should therefore be to discuss and deliberate on the ethical concerns of projects in a deliberative process to arrive at a conclusion that is most acceptable to the majority of stakeholders involved. This would require adopting consensus building in the decision-making to ensure that approved projects are acceptable in the face of all stakeholders involved in the process [35]. Furthermore, the guideline of Prentice and colleagues [53], the only aid that addressed consistency, did not include avenues for mathematical weighing of harms and benefits. This means attaining ethically consistent decision-making might not need to rely on the use of exact methods for evaluation.

The desired consistency of evaluations may be attainable only among similar types of research, while being difficult to achieve across various research fields. In order to realize this form of consistency, there may be a need for more research-specific decision aids to be developed and applied in specific contexts. The decision aid of Liguori and colleagues [39] for example, focused on research projects involving tissue engineering due to the absence of decision aids for reviewing projects of such specificity. Delpire and colleagues [42, 60] also adapted their decision aids for research involving transgenic animals. More of these decision aids could be developed to focus on specific experimental procedures to help ensure consistent evaluation of research projects adopting similar experimental procedures.

To add to these, an important point to consider is that the existence of these decision aids does not mean that they are used in practice. Generally, the decision aids were developed with specific purposes and they appear to be adequate in serving those purposes. This observation is supported by the comparison carried out by de Cock Buning and Theune [50] for three decision aids [47, 50, 56] proposed with the aim of serving different purposes. Nevertheless, we observed that most of the decision aids were developed by academic researchers as part of their research on animal research ethics. With these decision aids, it is difficult to determine the weight they carry in terms of the opportunity or obligation of oversight bodies to use them in their day-to-day project evaluations. For some of the decision aids [49, 50, 53, 57–59, 61], members of oversight bodies were among the authors. However, the certainty that those decision aids will be used by oversight bodies in their day-to-day was not established. Regarding decision aids of working groups, that of the EU Expert Working Group [33] convened by the EU, with member states nominating the members of the group, was recommended to assist member states in arriving at a common understanding of Directive 2010/63/EU, and to suggest ways in which the directive’s requirements can be met. This implies that the working group’s aid may guide project evaluations in member states. However, the other two decision aids proposed by working groups or parties [38, 56] do not come with clear recommendations or obligations for their application in project reviews. In essence, the proposal of these decision aids is a contribution to the collective effort of improving the decision-making process. The tools can inform oversight bodies regarding how they can conduct HBA, but the actual adherence to and application of most of the tools does not seem to have any form of obligatory enforcement. Therefore, their use is left to the decision of oversight authorities who may or may not use most of them in practice.

### Limitations

A limitation of our review is that grey literature was not searched since we only considered peer reviewed sources in the initial search. It is possible that some decision aids may only be identified through a grey literature search. However, we conducted a thorough screening of references of relevant sources of evidence captured by our search strategy at the full-text screening stage. Since some of these publications assessed decision-making processes in addition to known decision aids, we identified the known aids found in the grey literature. It is however possible that decision aids that are not peer reviewed may still be in the grey literature. Additionally, we considered only two publication languages (i.e., English and French) and decision aids in languages other than these two were not captured in our review.

## Conclusions

This Scoping Review has identified and described various proposed decision aids for the evaluation of experiments involving animals. Decision aids for HBA can take different approaches to ethical evaluation due to the varying ethical perspectives of the authors or groups proposing them. Ethical ideologies are also reflected in what is considered as harm to animals and what should count as benefit that justifies an animal research project. How a particular decision aid can be chosen to be used in oversight committees (which are generally composed of individuals with varying perspectives) can be challenging.

Moreover, the HBA concept is evolving over time, mainly in the search for terminology that clearly communicate the animal welfare concerns associated with animal research. Furthermore, proposed decision aids may be aiming for accurate and consistent decision-making, but these do not determine ethical acceptability of the decisions made with them. Since animal research projects can differ largely with respect to their ethical contents, it is essential for project reviews to focus on ethical case-by-case decision-making which can bring consistency over time. Furthermore, research conducted to understand how oversight bodies make decisions should also be done with respect to each experiments’ decision, and preferably not by looking across different experiments to come up with conclusions regarding the ethical acceptability of decisions made by oversight bodies. Focusing on ethically acceptable decisions for each experiment may result in consistent ethical decision-making over time as oversight committees can refer to previous ethical reviews to inform similar new reviews to attain some consistency. Nevertheless, the desired consistency can still be pursued among similar projects, and there may be the need for more research-specific decision aids to be developed for specific contexts to avoid a “one size fits all” scenario.

Finally, the aids contribute to the collective effort of improving the decision-making process as they can inform oversight bodies regarding how they can conduct HBA, but the decision to put them to use in evaluations remains with oversight authorities. More research need to be done among the members of review committees regarding the usefulness of these decision aids in practice.

## Supplementary Information

Supplementary Material 1.

Supplementary Material 2.

Supplementary Material 3.

Supplementary Material 4.

## Data Availability

No datasets were generated or analysed during the current study.

## Abbreviations

3Rs: Replacement, Refinement, and Reduction
AALAS: American Association for Laboratory Animal Science
FELASA: Federation of European Laboratory Animal Science Associations
HBA: Harm-Benefit Anaylsis
LASA: Laboratory Animal Science Association
PRISMA: Preferred Reporting Items for Systematic reviews and Meta-Analyses

## References

[1] Committee on the Use of Animals in Research - Councils of the National Academy of Sciences and the Institute of Medicine. Science, medicine, and animals. National Academies; 1991. 10.17226/10089.

[2] Brønstad A, Newcomer C, Decelle T, Everitt JI, Guillen J, Laber K. Current concepts of harm-benefit analysis of animal experiments - Report from the AALAS-FELASA working group on harm-benefit analysis - Part 1. Lab Anim. 2016;50:1–20. 10.1177/0023677216642398. 27188275 10.1177/0023677216642398 PMC5815836

[3] Olsson IAS, Hansen AK, Sandøe P. Animal welfare and the refinement of neuroscience research methods–a case study of Huntington’s disease models. Lab Anim. 2008;42:277–83. 10.1258/LA.2008.007147. 18625582 10.1258/la.2008.007147

[4] Ferdowsian HR, Beck N. Ethical and scientific considerations regarding animal testing and research. PLoS ONE. 2011;6:e24059. 10.1371/JOURNAL.PONE.0024059. 21915280 10.1371/journal.pone.0024059 PMC3168484

[5] The Principles of Humane Experimental Technique. Med J Aust. 1960;1:500–500. 10.5694/J.1326-5377.1960.TB73127.X.

[6] Hubrecht RC, Carter E. The 3Rs and humane experimental technique: implementing change. Animals. 2019. 10.3390/ANI9100754. 10.3390/ani9100754 PMC6826930 31575048

[7] Fitzpatrick BG, Koustova E, Wang Y. Getting personal with the reproducibility crisis: interviews in the animal research community. Lab Anim 2018. 2018;47:7. 10.1038/s41684-018-0088-6. 10.1038/s41684-018-0088-6 PMC7238286 29942056

[8] Akhtar A. The flaws and human harms of animal experimentation. Camb Q Healthc Ethics. 2015;24:407–19. 10.1017/S0963180115000079. 26364776 10.1017/S0963180115000079 PMC4594046

[9] Collins FS, Tabak LA, Policy. NIH plans to enhance reproducibility. Nature 2014 505:7485 2014;505:612–3. 10.1038/505612a 10.1038/505612a PMC4058759 24482835

[10] Green SB. Can animal data translate to innovations necessary for a new era of patient-centred and individualised healthcare? Bias in preclinical animal research. BMC Med Ethics. 2015;16:1–14. 10.1186/s12910-015-0043-7. 26215508 10.1186/s12910-015-0043-7 PMC4517563

[11] Begley CG, Ioannidis JPA. Reproducibility in science. Circ Res. 2015;116:116–26. 10.1161/CIRCRESAHA.114.303819. 25552691 10.1161/CIRCRESAHA.114.303819

[12] Wilson E, Ramage FJ, Wever KE, Sena ES, Macleod MR, Currie GL. Designing, conducting, and reporting reproducible animal experiments. J Endocrinol. 2023. 10.1530/JOE-22-0330. 10.1530/JOE-22-0330 PMC10304908 37074416

[13] MacLeod M, Mohan S. Reproducibility and rigor in animal-based research. ILAR J. 2019;60:17–23. 10.1093/ILAR/ILZ015. 31687758 10.1093/ilar/ilz015 PMC7275809

[14] Ioannidis JPA. Extrapolating from animals to humans. Sci Transl Med. 2012. 10.1126/SCITRANSLMED.3004631. 10.1126/scitranslmed.3004631 22972841

[15] Pound P, Ebrahim S, Sandercock P, Bracken MB, Roberts I. Where is the evidence that animal research benefits humans? BMJ. 2004;328:514–7. 10.1136/BMJ.328.7438.514. 14988196 10.1136/bmj.328.7438.514 PMC351856

[16] Bracken MB. Why are so many epidemiology associations inflated or wrong?? Does poorly conducted animal research suggest implausible hypotheses?? Ann Epidemiol. 2009;19:220–4. 10.1016/J.ANNEPIDEM.2008.11.006. 19217006 10.1016/j.annepidem.2008.11.006

[17] Würbel H. More than 3Rs: the importance of scientific validity for harm-benefit analysis of animal research. Lab Anim. 2017;46:164–6. 10.1038/laban.1220. 10.1038/laban.1220 28328898

[18] Eggel M, Würbel H. Internal consistency and compatibility of the 3Rs and 3Vs principles for project evaluation of animal research. Lab Anim. 2021;55:233–43. 10.1177/0023677220968583. 33215575 10.1177/0023677220968583 PMC8182293

[19] Vogt L, Reichlin TS, Nathues C, Würbel H. Authorization of animal experiments is based on confidence rather than evidence of scientific rigor. PLoS Biol. 2016;14:e2000598. 10.1371/journal.pbio.2000598. 27911892 10.1371/journal.pbio.2000598 PMC5135031

[20] Graham ML, Prescott MJ. The multifactorial role of the 3Rs in shifting the harm-benefit analysis in animal models of disease. Eur J Pharmacol. 2015;759:19–29. 10.1016/J.EJPHAR.2015.03.040. 25823812 10.1016/j.ejphar.2015.03.040 PMC4441106

[21] Fernandes MR, Pedroso AR. Animal experimentation: a look into ethics, welfare and alternative methods. Rev Assoc Med Bras. 2017;63:923–8. 10.1590/1806-9282.63.11.923. 29451652 10.1590/1806-9282.63.11.923

[22] European Commission. Directive 2010/63/EU of the European Parliament and of the Council of 22 September 2010 on the protection of animals used for scientific purposes. European Parliament; 2010.

[23] Schuppli CA. Decisions about the use of animals in research: ethical reflection by animal ethics committee members. Anthrozoos. 2011;24:409–25. 10.2752/175303711X13159027359980.

[24] Hansen LA, Goodman JR, Chandna A, Analysis of Animal Research Ethics Committee Membership at American Institutions. Animals 2012, Vol 2, Pages 68–75. 2012;2:68–75. 10.3390/ANI2010068 10.3390/ani2010068 PMC4494267 26486777

[25] Bout HJ, van Vlissingen JMF, Karssing ED. Evaluating the ethical acceptability of animal research. Lab Anim 2014. 2014;43:11. 10.1038/laban.572. 10.1038/laban.572 25333594

[26] Grimm H. Turning Apples into Oranges? The Harm-Benefit Analysis and how to Take Ethical Considerations into Account. Https://DoiOrg/101177/026119291504300211 2015;43:P22–4. 10.1177/026119291504300211 10.1177/026119291504300211 25995017

[27] Tjärnström E, Weber EM, Hultgren J, Röcklinsberg H. Emotions and Ethical Decision-Making in Animal Ethics Committees. Animals 2018, Vol 8, Page 181 2018;8:181. 10.3390/ANI8100181 10.3390/ani8100181 PMC6210494 30336584

[28] Varga O. Critical analysis of assessment studies of the animal ethics review process †. Animals. 2013;3:907. 10.3390/ANI3030907. 26479540 10.3390/ani3030907 PMC4494455

[29] Houde L, Dumas C, Leroux T. Animal ethical evaluation: an observational study of Canadian IACUCs. Ethics Behav. 2003;13:333–50. 10.1207/S15327019EB1304_2. 15000096 10.1207/S15327019EB1304_2

[30] Nordgren A, Röcklinsberg H. Genetically modified animals in research: an analysis of applications submitted to ethics committees on animal experimentation in Sweden. Anim Welf. 2005;14:239–48. 10.1017/S0962728600029407.

[31] Toulmin S. The tyranny of principles. Hastings Cent Rep. 1981;11:31. 10.2307/3560542. 7037683

[32] Bateson P. When to experiment on animals. New Sci. 1986;109:30–2. 11655736

[33] Expert Working Group for Project Evaluation and Retrospective Assessment. Caring for animals aiming for better science: Directive 2010/63/EU on protection of animals used for scientific purposes: project evaluation and retrospective assessment: Working document on Project Evaluation and Retrospective Assessment. 2018. 10.2779/59814

[34] Jörgensen S, Lindsjö J, Weber EM, Röcklinsberg H. Reviewing the review: a pilot study of the ethical review process of animal research in Sweden. Anim (Basel). 2021;11:1–28. 10.3390/ANI11030708. 10.3390/ani11030708 PMC8002130 33807898

[35] Azilagbetor DM, Shaw D, Elger BS. Animal Research Regulation: Improving Decision-Making and Adopting a Transparent System to Address Concerns around Approval Rate of Experiments. Animals 2024, Vol 14, Page 846. 2024;14:846. 10.3390/ANI14060846 10.3390/ani14060846 PMC10967461 38539944

[36] Grimm H, Eggel M, Deplazes-Zemp A, Biller-Andorno N. The road to hell is paved with good intentions: why harm–benefit analysis and its emphasis on practical benefit jeopardizes the credibility of research. Animals. 2017;7:70. 10.3390/ani7090070. 28892015 10.3390/ani7090070 PMC5615301

[37] Davies GF. Harm-Benefit Analysis: opportunities for enhancing ethical review in animal research. Lab Animal. 2018 47:3 2018;47:57–8. 10.1038/s41684-018-0002-2 10.1038/s41684-018-0002-2 29483693

[38] Laber K, Newcomer CE, Decelle T, Everitt JI, Guillen J, Brønstad A. Recommendations for addressing harm–benefit analysis and implementation in ethical evaluation – report from the AALAS–FELASA working group on harm–benefit analysis – part 2. Lab Anim. 2016;50:21–42. 10.1177/0023677216642397. 10.1177/0023677216642397 PMC5815834 27188276

[39] Liguori GR, Jeronimus BF, De Aquinas Liguori TT, Moreira LFP, Harmsen MC. Ethical issues in the use of animal models for tissue engineering: reflections on legal aspects, moral theory, three Rs strategies, and harm’benefit analysis. Tissue Engineering Part C: Methods. 2017;23:850–62. 10.1089/TEN.TEC.2017.0189. 28756735 10.1089/ten.TEC.2017.0189

[40] Smith JA, Van Den Broek FAR, Martorell JC, Hackbarth H, Ruksenas O, Zeller W. Principles and practice in ethical review of animal experiments across europe: summary of the report of a FELASA working group on ethical evaluation of animal experiments. Lab Anim. 2007;41:143–60. 10.1258/002367707780378212/SUPPL_FILE/FELASA_ETHICS.PDF. 17430615 10.1258/002367707780378212

[41] Grimm H, Olsson IAS, Sandøe P. Harm–benefit analysis – what is the added value? A review of alternative strategies for weighing harms and benefits as part of the assessment of animal research. Lab Anim. 2019;53:17–27. 10.1177/0023677218783004/ASSET/IMAGES/LARGE/10.1177_0023677218783004-FIG2.JPEG. 29966482 10.1177/0023677218783004

[42] Delpire VC, Mepham T, Ben, Balls M. A Proposal for a New Ethical Scheme Addressing the Use of Laboratory Animals for Biomedical Purposes. Https://DoiOrg/101177/026119299902701s01 1999;27:869–81. 10.1177/026119299902701S01

[43] Talbot SR, Struve B, Wassermann L, Heider M, Weegh N, Knape T, et al. RELSA—A multidimensional procedure for the comparative assessment of well-being and the quantitative determination of severity in experimental procedures. Front Vet Sci. 2022;9:937711. 10.3389/FVETS.2022.937711/BIBTEX. 36439346 10.3389/fvets.2022.937711 PMC9691969

[44] Mieske P, Hobbiesiefken U, Fischer-Tenhagen C, Heinl C, Hohlbaum K, Kahnau P, et al. Bored at home?—A systematic review on the effect of environmental enrichment on the welfare of laboratory rats and mice. Front Vet Sci. 2022;9:899219. 10.3389/FVETS.2022.899219/BIBTEX. 36061113 10.3389/fvets.2022.899219 PMC9435384

[45] Tappe-Theodor A, Pitzer C, Lewejohann L, Jirkof P, Siegeler K, Segelcke A, et al. The wwhow concept for prospective categorization of post-operative severity assessment in mice and rats. Front Vet Sci. 2022;9:841431. 10.3389/FVETS.2022.841431. 35372532 10.3389/fvets.2022.841431 PMC8964947

[46] Quimby FW. Twenty-five years of progress in laboratory animal science. Lab Anim. 1994;28(2):158–71. 10.1258/002367794780745335. 8035568 10.1258/002367794780745335

[47] Porter DG. Ethical scores for animal experiments. Nature. 1992;356:101–2. 10.1038/356101a0. 1545854 10.1038/356101a0

[48] Stafleu FR, Tramper R, Vorstenbosch J, Joles JA. The ethical acceptability of animal experiments: proposal for a system to support decision-making. Lab Anim. 1999;33:295–303. 10.1258/002367799780578255. 10780850 10.1258/002367799780578255

[49] Voipio H-M, Kaliste E, Hirsjärvi P, Nevalainen T, Ritskes-Hoitinga M. Nordic-European workshop on ethical evaluation of animal experiments. Scand J Lab Anim Sci. 2004;31:251–67.

[50] de Cock Buning T, Theune EP. A comparison of three models for ethical evaluation of proposed animal experiments. Anim Welf. 1994;3:107–28. 10.1017/S0962728600016614. 11660271

[51] Tricco AC, Lillie E, Zarin W, O’Brien KK, Colquhoun H, Levac D, et al. PRISMA extension for scoping reviews (PRISMA-ScR): checklist and explanation. Ann Intern Med. 2018;169:467–73. 10.7326/M18-0850. 30178033 10.7326/M18-0850

[52] Azilagbetor DM, Morales RMC, Shaw DM, Geneviève LD, Elger BS, Gaab J. Decision aids for harm-benefit analyses (HBA) conducted in animal research evaluations: A scoping Review Protocol 2023. 10.17605/OSF.IO/PTMWJ

[53] Prentice ED, Crouse DA, Rings RW, Prentice ED. Approaches to increasing the ethical consistency of prior review of animal research. Invest Radiol. 1990;25:271–4. 10.1097/00004424-199003000-00012. 2332313 10.1097/00004424-199003000-00012

[54] Niemi SM. Harm-benefit analyses can be harmful. ILAR J. 2019;60:341–6. 10.1093/ILAR/ILAA016. 10.1093/ilar/ilaa016 32785593

[55] Gutfreund Y. Should Be Explored Anim. Harm-Benefit Analysis May Not Bethe Best Approach to Ensure Minimal Harms and Maximal Benefits of Animal Research—Alternatives. 2020;10:291. 10.3390/ani10020291. 10.3390/ani10020291 PMC7070552 32059540

[56] Smith JA, Boyd KM. The assessment and weighing of costs and benefits. In: Lives in the balance: the ethics of using animals in biomedical research: the report of a working party of the Institute of medical ethics. New York: Oxford University Press; 1991.

[57] Boisvert DP, Porter DG. Ethical scoring systems. In: Johnston NE, editor. Animal Welfare Conference Proceedings, Clayton, Victoria: Monash University; 1993, pp. 23–7.

[58] Animal Procedures Committee. Report of the Animal Procedures Committee for 1993. London: 1994.

[59] Boisvert DP, Porter DG. Ethical scoring systems. In: Goldberg AM, van Zutphen LFM, editors. Alternative methods in toxicology and the life sciences, vol. 11, the world Congress on alternatives and animal use in the life sciences: education, research. Testing, New York: Mary Ann Liebert; 1995. pp. 637–41.

[60] Delpire V, Shaw D, Crilly RE, Mepham TB, Combes RD, Balls M. A comprehensive evaluation of procedures involving the use of Transgenic animals within the European union. In: O’Donoghue PN, editor. The ethics of animal experimentation. London: EBRA; 1998. pp. 223–8.

[61] Lindl T, Gross U, Ruhdel I, Von Aulock S, Völkel M. Guidance on determining indispensability and balancing potential benefits of animal experiments with costs to the animals with specific consideration of EU directive 2010/63/EU. ALTEX - Alternatives to. Anim Experimentation. 2012;29:219–28. 10.14573/ALTEX.2012.2.219. 10.14573/altex.2012.2.219 22562491

[62] Mellor DJ. Comprehensive assessment of harms caused by experimental, teaching and testing procedures on live animals. Altern Lab Anim. 2004;32:453–7. 10.1177/026119290403201S73. 23581117 10.1177/026119290403201s73

[63] Mellor D, Reid C. Concepts of animal well-being and predicting the impact of procedures on experimental animals. In: Baker R, Jenkin G, Mellor D, editors. Improving the well-being of animals in the research environment, Australia: Australian and New Zealand Council for the Care of Animals in Research and Teaching, 1994, pp. 3–18.

[64] Wallace J, Sanford J, Smith MW, Spencer KV. The assessment and control of the severity of scientific procedures on laboratory animals: report of the laboratory animal science association working party (Assessment and control of severity). Lab Anim. 1990;24:97–130. 10.1258/002367790780890185/ASSET/002367790780890185.FP.PNG_V03. 2366519 10.1258/002367790780890185

[65] Ferreira GS, Veening-Griffioen DH, Boon WPC, Moors EHM, De Wied CCG, Schellekens H, et al. A standardised framework to identify optimal animal models for efficacy assessment in drug development. PLoS ONE. 2019;14:e0218014. 10.1371/JOURNAL.PONE.0218014. 31194784 10.1371/journal.pone.0218014 PMC6563989

[66] Storey J, Gobbetti T, Olzinski A, Berridge BR. A structured approach to optimizing animal model selection for human translation: the animal model quality assessment. ILAR J. 2021;62:66–76. 10.1093/ILAR/ILAC004. 35421235 10.1093/ilar/ilac004 PMC9291347

[67] Baker HB, McQuilling JP, King NMP. Ethical considerations in tissue engineering research: case studies in translation. Methods. 2016;99:135–44. 10.1016/J.YMETH.2015.08.010. 26282436 10.1016/j.ymeth.2015.08.010 PMC4869966

[68] Mohan S, Foley PL. Everything you need to know about satisfying IACUC protocol requirements. ILAR J. 2019;60:50–7. 10.1093/ILAR/ILZ010. 31361817 10.1093/ilar/ilz010 PMC7304469

[69] Hall MR, Dardik A. Getting your IACUC proposal approved. In: Kibbe M, LeMaire S, editors. Success in academic surgery: basic science. Success in academic surgery. London: Springer; 2014. pp. 179–93. 10.1007/978-1-4471-4736-7_13.

[70] DeGrazia D. The moral status of animals and their use in research: a philosophical review. Kennedy Inst Ethics J. 1991;1:48–70. 10.1353/KEN.0.0112. 11645700 10.1353/ken.0.0112

[71] Foëx BA. The ethics of animal experimentation. Emerg Med J. 2007;24:750. 10.1136/EMJ.2007.050146. 17954822 10.1136/emj.2007.050146 PMC2658312

[72] Bakhle YS. Missing evidence that animal research benefits humans: evidence is all around us. BMJ: Br Med J. 2004;328:1017. 10.1136/BMJ.328.7446.1017-B. 10.1136/bmj.328.7446.1017-b PMC404545 15105339

[73] Blakemore C, Peatfield T. Missing evidence that animal research benefits humans: moratorium is unjustified. BMJ: Br Med J. 2004;328:1017. 10.1136/BMJ.328.7446.1017-C. 10.1136/bmj.328.7446.1017-c PMC404546 15105340

[74] Chang MCJ, Grieder FB. The continued importance of animals in biomedical research. Lab Animal. 2024 53:11 2024;53:295–7. 10.1038/s41684-024-01458-4 10.1038/s41684-024-01458-4 PMC11518981 39402213

[75] Abbott A. Basel declaration defends animal research. Nature. 2010;468:742. 10.1038/468742A. 21150964 10.1038/468742a

[76] Schiermeier Q. German authority halts primate work. Nature. 2008;455:1159. 10.1038/4551159A. 18985863 10.1038/4551159a

[77] Abbott A. Swiss court bans work on macaque brains. Nature. 2008;453:833. 10.1038/453833A. 18548030 10.1038/453833a

[78] Kinter LB, Johnson DK. A defense of risk-benefit terminology. Lab Animal. 2015 44:10 2015;44:403–7. 10.1038/laban.875 10.1038/laban.875 26398615

[79] Veening-Griffioen DH, Ferreira GS, Boon WPC, Gispen-De Wied CC, Schellekens H, Moors EHM, et al. Tradition, not science, is the basis of animal model selection in translational and applied research. ALTEX - Alternatives to Animal Experimentation. 2021;38:49–62. 10.14573/ALTEX.2003301 10.14573/altex.2003301 32591838

