# Supplementary material for: A scoping review of ethical decisions and decision tools for experimental animal protocols
Source: BMC Med Ethics. 2025 Nov 14;26:160. doi: 10.1186/s12910-025-01297-z (PMC12619158; doi:10.1186/s12910-025-01297-z)
Supplement: Supplementary file 1 — Supplementary Material 1. [file 12910_2025_1297_MOESM1_ESM.docx]

**Search Strategy for PubMed**

| PubMed | ("Animal experimentation"[Mesh] OR ("Animal experiment"[tiab] OR "Animal research"[tiab] OR "animal testing"[tiab]))  AND  ("Ethics, Research"[Mesh] OR ("animal ethics committee*"[tiab] OR "animals in science committee*"[tiab] OR "animal care and use committee*"[tiab] OR "Institutional Animal Care and Use Committee*"[tiab]) OR ("harm-benefit analys*"[tiab] OR "harm-benefit assessment*"[tiab] OR "protocol assessment*"[tiab] OR "protocol evaluation*"[tiab] OR "weighing of interest*"[tiab] OR "benefit evaluation*"[tiab] OR "benefit assessment*"[tiab] OR "animal welfare"[tiab] OR "harm evaluation*"[tiab] OR "harm assessment*"[tiab])) |
| --- | --- |

**Data Charting Table**

| Publication characteristics | | | | | |
| --- | --- | --- | --- | --- | --- |
|  | **Item** | | **Question/Explanation** | **Example(s) if applicable** | |
| 1 (a) | Author(s) | |  |  | |
| 1 (b) | Title | |  |  | |
| 2 (a) | Year of Publication | |  |  | |
| 2 (b) | Aim of Publication | |  |  | |
|  | **Item** | | **Question/Explanation** | **Example(s) if applicable** | |
| 3 | Origin | Publication’s country | Geographical source:  Where is the decision aid developed or published?  Which country are the proposing authors in/from? | France, Belgium, Netherlands, U.S. and Canada | |
|  |  | Region of decision aid | In which region is the decision aid supposed to be applied? | Europe or North America | |
| 4 | Jurisdiction: Where is the decision aid supposed to be applied? | National | In which country is the decision aid supposed to be applied? | Decision aids applicable in France, Belgium, Netherlands, U.S. and Canada. | |
|  |  | Supra-national | In which supra-national jurisdiction is the decision aid supposed to be applied? | Decision aids applicable in the EU | |
| 5 | Proponents of the decision aid | | Who proposed/designed the decision aid? | i. Academia: Some researchers from academia developed decision aids as part of their own research. ii. Institutional: Institutional Animal Ethics Commissions like IACUCs, etc. iii. Working Group: A group of experts coming together to propose a decision aids. iv. Authority: An appointed group that has the competence to propose a decision aid (or a guideline) for decision-making in a specific jurisdiction. E.g.: Animal Procedures Committee in the UK.  (v) Non-Profit Organization (e.g. Charitable foundation; NGO, etc.) | |
| 6 | Approach to HBA process | | What methods do the aids adopt in reaching an HBA decision? | • Algorithms / Metric / Scoring: The use of mathematical formulae for HBA decision-making. • Graphic representations: Graphical visualization to guide HBA decision-making • Process-oriented models / Discourse models: An exchange among committee members in the form of discussions to reach a final decision • Categories: Categorization of harm and benefit items to consider in HBA decision-making • Combined (mixed) models: The use of more than one of the models above in HBA decision-making. | |
| Harms | | | | | |
|  | **Item** | | **Question/Explanation** | **Example(s) if applicable** | |
| 7 | Definition | | Is there a specific definition of harm provided? |  | |
|  | Constituents of harm discussed | | What is discussed in terms of harm or “costs”? | Pain  Discomfort (e.g. related to experiment or housing, etc.)  Suffering  Freedom limitations  Etc. | |
|  | Harm examples | | What examples of harm are provided? | Insertion of hypodermic needle  Surgery  Toxicity tests  Etc. | |
|  | Harm Assessment | | How can “harm” or its constituents be assessment?/ What tools/guidelines are provided to assess harm? | Discomfort assessment= level of discomfort (refer to directive + consider species-specific physiological and behavioral parameters) + frequency of operation + duration of discomfort  Etc.  Scoring system: ordinal (e.g. - to ++++), numerical (e.g. 0 to 10) or color | |
|  | Types of harm | | What are the types of harm considered in the decision-aid? | Physical  Psychological  Physiological  Social well-being  Negative results  Intrinsic value  Etc. | |
|  | Harm severity categorizations | | What harm severity categories are provided? | Minor/moderate/severe  Non-recovery/mild/moderate/severe | |
|  | Harm severity category delineations | | How or which tool is used to delineate the harm severity categories? | Definitions provided by Directive 2010/63 Annex VIII:  e.g. Non-recovery: procedures which are performed entirely under general anesthesia from which the animal shall not recover consciousness | |
| Benefits | | | | | |
|  | **Item** | | **Question/Explanation** | **Example(s) if applicable** | |
| 8 | Definition | | Is there a specific definition of benefit provided? |  | |
|  | Constituents of benefits discussed | | What is discussed in terms of benefits? | Scientific value  Social value  Economic value  Educational value  Etc. | |
|  | Areas of interests/purpose of research | |  | Basic research  Education  Applied research  Regulatory testing  Etc. | |
|  | Benefit examples | | What examples of benefits are provided? | Production of vaccines  New diagnostics  Etc. | |
|  | Benefit assessment | | How can “benefit” or its constituents be assessed? / What tools/guidelines are provided to assess them in the decision aid? | Use of a checklist  Use of a specific guideline  Scoring system: ordinal (e.g. - to ++++), numerical (e.g. 0 to 10) or color (e.g. low – red, intermediate – amber, and high - green) | |
|  | Benefit significance categorizations | | What benefit significance categories are provided? | Minor/moderate/great  Small/medium/large  Low, intermediate and high | |
|  | Benefit significance category delineations | | How or which tool is used to delineate the benefit significance categories? |  | |
| Harm-benefit balancing | | | | | |
|  | **Item** | | **Question/Explanation** | **Example(s) if applicable** | |
| 9 | i. How is harm balanced with benefit to reach a final conclusion? | | How is harm balanced with benefit to reach a final conclusion? |  | |
|  | ii. What tools are provided for this balancing exercise? | | What tools are provided for this balancing exercise? |  | |
|  | iii. Applicability of tool by oversight bodies | | Additional notes about the applicability of the tool by decision-making authorities |  | |
| Advantages and Limitations | | | | | |
|  | **Item** | | **Question/Explanation** | **Example(s) if applicable** | |
| 10 | Advantages of decision aids reported by the authors | | What advantages does the authors discuss?  What advantages can be observed? |  | |
| 11 | Limitations of decision aid: | | Have the authors cited any limitations (other than consistency) about their decision aids? |  | |
| Consistency | | | | | |
|  | **Item** | | **Question/Explanation** | | **Example(s) if applicable** |
| 12 | i. How do the decision-aids allow for a consistent evaluation? | | How do the decision-aids allow for a consistent evaluation? | |  |
|  | ii. What are specific limitations related to consistency? | | What are specific limitations related to consistency? | |  |
|  | iii. Have some of them led to decisional conflict? | | Have some of them led to decisional conflict? | |  |
