# Supplementary material for: A scoping review of ethical decisions and decision tools for experimental animal protocols
Source: BMC Med Ethics. 2025 Nov 14;26:160. doi: 10.1186/s12910-025-01297-z (PMC12619158; doi:10.1186/s12910-025-01297-z)
Supplement: Supplementary file 2 — Supplementary Material 2. [file 12910_2025_1297_MOESM2_ESM.docx]

**Characteristics of included publications**

| **Author(s)** | **Title** | **Year of Publication** | **Type of Publication** | **Aim of Publication** | **Category of Proponent** |
| --- | --- | --- | --- | --- | --- |
| Patrick Bateson [32] | When to experiment on animals | 1986 | Magazine article | To resolve the conflicts of interest between experimenters and their critics by weighing up the degree of suffering against the value of the research. | Academia |
| Ernest D. Prentice, David A. Crouse, Reed W. Rings [53] | Approaches to Increasing the Ethical Consistency of Prior Review of Animal Research | 1990 | Journal article | To present and discuss a protocol review guide which has contributed to consistent decision-making of the IACUCs at Nebraska Medical Center and its sister campus, the University of Nebraska at Omaha. | Institutional: IACUC |
| Jane A. Smith & Kenneth M. Boyd [56] | Lives in the Balance: The Ethics of Using Animals in Biomedical Research (Chapter 7: The assessment and 'weighing' of costs and benefits) | 1991 | Book chapter | To draw up assessment schemes to assist in determining the potential harm (or cost) imposed on animals used in biomedical research as well as to determine the potential benefits such research. | Non-Profit Organization (Charitable Foundation) |
| David G. Porter [47] | Ethical scores for animal experiments | 1992 | Journal article | To propose a simple scoring system to explore animal experiments from the perspective of the research animal. | Academia |
| Donald P. Boisvert & David G. Porter [57] | Ethical scoring systems. (In: Animal Welfare Conference  Proceedings (ed. N.E. Johnston)) | 1993 | Conference proceeding | Revising the proposal of D.G Porter (1992) to provide a version that could be applied by the Canadian Animal Care Committees (ACC) in evaluating of animal research protocols. | Academia and Authority |
| Tj de Cock Buning & E Theune (Dutch model) [50] | A comparison of three models for ethical evaluation of proposed animal experiments | 1994 | Journal article | To compare and discuss three models used in animal research evaluations: (i) a Dutch model, (ii) the Porter Model (Canadian), (iii) the British IME model. | Academia and Authority |
| Animal Procedures Committee [58] | Report of the Animal Procedures Committee for 1993 | 1994 | Report of statutory body | Report of the Animal Procedures Committee which is tasked to advise the Home Secretary on matters regarding the Animal (Scientific Procedures) Act 1986. | Authority |
| Donald P. Boisvert & David G.Porter [59] | Ethical scoring systems. (In: Alternative Methods in Toxicology and the Life Sciences, Vol. 11, The World Congress on Alternatives and Animal Use in the Life Sciences: Education, Research, Testing (ed. A.M. Goldberg & L.F.M. van Zutphen)) | 1995 | Conference proceeding | To revise the proposal of D.G Porter (1992) to produce an ethical check-list usable by the Canadian Animal Care Committees in evaluating animal research protocols. | Academia and Authority |
| Delpire, V., Shaw, D., Crilly, R.E., Mepham,  T.B., Combes, R.D. & Balls, M [60] | A comprehensive evaluation of procedures involving the use of transgenic animals within the European Union. (In: The Ethics of Animal Experimentation (ed. P.N. O’Donoghue)) | 1998 | Conference proceeding | To offer a thorough analysis of the scientific and ethical concerns about the use of transgenic animals in the European Union and propose a scheme to facilitate the assessment and evaluation of ethical issues relating to the use of transgenic animals. | Academia |
| Véronique C. Delpire, T. Ben Mepham and Michael Balls [42] | A Proposal for a New Ethical Scheme Addressing the Use of Laboratory Animals for Biomedical Purposes | 1999 | Journal article | To discuss a new ethical scheme for evaluating animal experiment projects in the biomedical sciences, including those involving transgenic animals, in Europe. | Academia |
| F. R. Stafleu, R. Tramper, J. Vorstenbosch & J. A. Joles [48] | The ethical acceptability of animal experiments: a proposal for a system to support decision-making | 1999 | Journal article | To describe a system to assist in a systematic ethical decision-making done by researchers, members of animal experiments review committees, on animal experiments. | Academia |
| Hanna-Marja Voipio, Eila Kaliste, Paula Hirsjärvi, Timo Nevalainen, Merel Ritskes-Hoitinga [49] | Nordic-European workshop on ethical evaluation of animal experiments | 2004 | Journal article | A paper from a workshop aimed at comparing animal research evaluation in each participating countries, to review their methods for ethical evaluation, and come to a consensus on conducting ethical evaluations. | Academia, Institutional and Authority |
| Toni Lindl, Ulrike Gross, Irmela Ruhdel, Sonja von Aulock, and Manfred Völkel [61] | Guidance on Determining Indispensability and Balancing Potential Benefits of Animal Experiments with Costs to the Animals with Specific Consideration of EU Directive 2010/63/EU | 2012 | Journal article | A report of a workshop that aimed to reach transparent and easily comprehensible decisions and to propose a formal procedure that simplifies the evaluation of animal experiment applications in the authorization process. | Academia, Institutional and Authority |
| Henriëtte J. Bout, J. Martje Fentener van Vlissingen &  Edgar D. Karssing [25] | Evaluating the ethical acceptability of animal research | 2014 | Journal article | To assess different ethics models and observe some ethics commissions to propose a matrix for carrying out harm-benefit analyses. | Academia |
| Kathy Laber, Christian E Newcomer, Thierry Decelle, Jeffrey I Everitt, Javier Guillen, and Aurora Brønstad [38] | Recommendations for Addressing Harm-Benefit Analysis and Implementation in Ethical Evaluation - Report from the AALAS-FELASA Working Group on Harm-Benefit Analysis - Part 2 | 2016 | Journal article | To present a method for conducting HBA proposed by the AALAS-FELASA working group on HBA. | Working Group |
| Gabriel R. Liguori, Bertus F. Jeronimus, Tácia T. de Aquinas Liguori, Luiz Felipe P. Moreira, and Martin C. Harmsen [39] | Ethical Issues in the Use of Animal Models for Tissue Engineering: Reflections on Legal Aspects, Moral Theory, Three Rs Strategies, and Harm–Benefit Analysis | 2017 | Journal article | To provide viewpoints for implementing minimum acceptable standards for animal experimentation in Europe and USA, and propose a tool for conducting HBA in the context of tissue engineering (TE). | Academia |
| EU Expert Working Group [33] | Caring for animals aiming for better science: Directive 2010/63/EU on protection of animals used for scientific purposes : project evaluation and retrospective assessment: Working document on Project Evaluation and Retrospective Assessment | 2018 | Working document | A working document of an expert working group to facilitate the implementation of Directive 2010/63/EU in evaluating animal research projects and conducting retrospective assessments. | Working Group |
