# Supplementary material for: A scoping review of ethical decisions and decision tools for experimental animal protocols
Source: BMC Med Ethics. 2025 Nov 14;26:160. doi: 10.1186/s12910-025-01297-z (PMC12619158; doi:10.1186/s12910-025-01297-z)
Supplement: Supplementary file 3 — Supplementary Material 3. [file 12910_2025_1297_MOESM3_ESM.docx]

**The issue of consistency addressed by decision aids**

| **Decision Aid (Authors/Year)** | **How Does/Can it Allow for Consistent Evaluations** | **Specific Limitations to Consistency** | **Has it Led to Decisional Conflict?** |
| --- | --- | --- | --- |
| Bateson, 1986 [32] | Use of the decision cube | Subjectivity in deciding the level to be assigned to the three components of the decision cube | Not discussed |
| Prentice et al., 1990 [53] | Found to reduce variability and improve consistency due to the use of the protocol review form and protocol review guide which provides the informational base for the review and allow members to sequentially apply the same evaluative items. | None discussed | Not discussed |
| Smith & Boyd, 1991 [56] | The use of the schemes provided, assessing different aspects of the proposed animal experiment, and culminating in the categorical evaluation (low, medium and high) of the overall costs and benefits for decision-making. | 1) No clear guidance on the overall weighing of costs against benefits to reach a final decision; 2) Decision-making is based on subjective judgements of committee members, and there may be variations in case-by-case decision-making. | Not discussed |
| Porter, 1992 [47] | The use of its categorical scoring system. | None discussed | Not discussed |
| Boisvert & Porter, 1993 [57] | By the application of its seemingly exactness approach. | May be subjective, not suitable for research other than those involving captive animals, introduces bias against basic research, and may therefore lead to different results. | Not discussed |
| de Cock Buning & Theune, 1994 [50] | The use of an algorithm (decision tree) and a comprehensive checklist containing all the relevant questions. | 1) Committee members having to determine on their own, under which category main interests of the project fall, can be problematic in projects where there are no clear boundaries (i.e. projects having both health and scientific interest); 2) Burden of inter-committee jurisprudence and dialogue on how heavy unsatisfactory aspects of the research will weigh in their judgement for consistency. | Not discussed |
| Animal Procedures Committee, 1994 [58] | Does not allow for a consistent decision-making, even with the use of the mathematical formula (see limitation). | Subjective judgement on the benefits and costs. | Not discussed |
| Boisvert & Porter, 1995 [59] | The use of checklist may offer some consistency in decision-making. | Subjectivity in assigning scores/values to the overall assessment of benefits and costs. | Not discussed |
| Delpire et al., 1998 [60] | The series of questions to be scored in the scheme can guide consistent decision-making. | Subjectivity in assigning scores, including how the aggregated score is to be assigned any of the four evaluation outcomes. | Not discussed |
| Delpire et al., 1999[42] | The use of checklist and explanatory guidance notes serving as a common framework in the EU can standardize decision-making, ensuring consistency. Its focus on transgenic animals which are widely used in research can provide harmony and consistency for most animal experiments. | 1) Leaving each committee to subjectively decide on criteria and determine the completeness of information could result in inconsistent decision-making among different committees; 2) Neither defines harm nor provides guidance on how harm can be measured. It does not also give a system of classification of harm. | Not discussed |
| Stafleu et al., 1999 [48] | Algorithm and formulas to be used in decision-making. Its seemingly objective scoring of harm and benefits can lead to consistent decisions. | Not discussed | Not discussed |
| Voipio et al., 2004 [49] | Provision of checklists for benefits, costs and cost modifiers. | Scoring of costs and benefits are subjective and may bring differences in study-to-study evaluations, even though their goals may remain the same. | Large inter-individual variation observed for the scoring of harm, benefits and means. |
| Lindl et al., 2012 [61] | Provides a comprehensive and sequential procedure to be followed in the ethical assessment. | Not discussed | Not discussed |
| Bout et al., 2014 [25] | Provision of a graphical matrix. How it can allow consistent decision-making is however not discussed. | Subjectivity in assessing potential benefits. | Not discussed |
| Laber et al., 2016 [38] | 1) Provision of a template that helps to standardize the assessment approach in HBA processes; 2) A list of definitions is provided for each modulating factor. | 1) Inherent challenges in assessing severity, warranting professional judgment; 2) Some elements contributing to decision-making cannot be accounted for by the tool: moral consciences and value judgments of individuals concerning harm and benefits for a specific project. | Not discussed |
| Liguori et al., 2017 [39] | 1) A flow chart added to a graphic representation of the algorithmic HBA that provide a roadmap for the balancing of benefits and harm, including a scoring system for the translational gap; 2) Adopts objective and scientifically rigorous model and could ensure consistency. | 1) No definitions of what constitutes short/long-terms animal pain/harm; 2) Challenges of inconsistency due to the prominent role of the selected animal model which can render it difficult to meet all algorithm requirements; 3) Additional considerations in the field of TE, may require other components to be added to this decision aid to maintain its objectivity, operability and consistency. | Not discussed |
| EU Expert Working Group, 2018 [33] | Not discussed | Subjectivity is an unavoidable component of the analysis; a balanced constitution of competent assessors and a structured approach to ensure consistency, is needed. | Not discussed |
